# Elaboration of *Trans*-Resveratrol Derivative-Loaded Superparamagnetic Iron Oxide Nanoparticles for Glioma Treatment

**DOI:** 10.3390/nano9020287

**Published:** 2019-02-18

**Authors:** Fadoua Sallem, Rihab Haji, Dominique Vervandier-Fasseur, Thomas Nury, Lionel Maurizi, Julien Boudon, Gérard Lizard, Nadine Millot

**Affiliations:** 1 Laboratoire Interdisciplinaire Carnot de Bourgogne (ICB), UMR 6303 CNRS/Université Bourgogne Franche-Comté, 21000 Dijon, France; fadouasallem@gmail.com (F.S.); lionelmaurizi@gmail.com (L.M.); julien.boudon@u-bourgogne.fr (J.B.); 2 Institut de Chimie Moléculaire de l’Université de Bourgogne (ICMUB), UMR 6302 CNRS/Université Bourgogne Franche-Comté, 21000 Dijon, France; hajirihab22@yahoo.com (R.H.); dominique.vervandier-fasseur@u-bourgogne.fr (D.V.-F.); 3 Laboratoire Bio-PeroxIL, EA7270, Université de Bourgogne Franche-Comté/Inserm, 21000 Dijon, France; thomas.nury@u-bourgogne.fr (T.N.); gerard.lizard@u-bourgogne.fr (G.L.)

**Keywords:** iron oxide superparamagnetic nanoparticles, *trans*-resveratrol derivative, drug delivery, glioma

## Abstract

In this work, new nanohybrids based on superparamagnetic iron oxide nanoparticles (SPIONs) were elaborated and discussed for the first time as nanovectors of a derivative molecule of trans-resveratrol (RSV), a natural antioxidant molecule, which can be useful for brain disease treatment. The derivative molecule was chemically synthesized (4’-hydroxy-4-(3-aminopropoxy) trans-stilbene: HAPtS) and then grafted onto SPIONs surface using an organosilane coupling agent, which is 3-chloropropyltriethoxysilane (CPTES) and based on nucleophilic substitution reactions. The amount of HAPtS loaded onto SPIONs surface was estimated by thermogravimetric analysis (TGA) and X-ray photoelectron spectroscopy (XPS) analyses at 116 µmol·g^−1^ SPIONs. The synthesized HAPtS molecule, as well as the associated nanohybrids, were fully characterized by transmission electron microscopy (TEM), XPS, TGA, infrared (IR) and UV-visible spectroscopies, dynamic light scattering (DLS), and zeta potential measurements. The in vitro biological assessment of the synthesized nanohybrid’s efficiency was carried out on C6 glioma cells and showed that the nanovector SPIONs-CPTES-HAPtS do not affect the mitochondrial metabolism (MTT test), but damage the plasma membrane (FDA test), which could contribute to limiting the proliferation of cancerous cells (clonogenic test) at a HAPtS concentration of 50 µM. These nanoparticles have a potential cytotoxic effect that could be used to eliminate cancer cells.

## 1. Introduction

**Figure 1:**
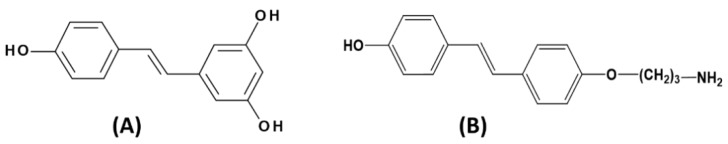
Chemical structure of (**A**) the natural trans-resveratrol molecule (RSV) and (**B**) the trans-resveratrol derivative molecule: 4’-hydroxy-4-(3-aminopropoxy) trans-stilbene (HAPtS).

Trans-resveratrol, or 3,4’,5-trihydroxy-trans-stilbene (RSV) (Figure 1A), is a polyphenolic compound that belongs to the stilbene family. It is widespread in the plant kingdom and found especially in peanuts, grapes, and accordingly, in wine [1,2]. The discovery of RSV in red wine and interest in its ability to prevent cardiovascular diseases was the starting point of this molecule [3]. Since then, numerous in vitro and in vivo studies in animals have shown various biological properties of RSV, such as antioxidant [4], anti-microbial [5], anti-inflammatory [6], estrogenomimetic effects, anti-cancer [7], and chemopreventive activities [8]. Indeed, thanks to its potent antioxidant power, it has proven its effectiveness against skin, breast, lung, prostate, and pancreas cancers [9].

As oxidative damage has been considered the main cause of many neurodegenerative diseases, including Alzheimer’s disease (AD), Parkinson’s disease (PD), and stroke [10,11], RSV has extensively been studied as a therapeutic molecule for these kinds of diseases based on its antioxidant properties [12]. In fact, the neuroprotective effects of RSV against oxidative stress was proved by R. Alyssa et al. [13]. Moreover, a study by Wang et al. showed that RSV can cross the blood brain barrier (BBB) and protect against cerebral ischemic injury [14]. RSV was also shown to inhibit the formation of amyloid-beta (Aβ) aggregation characterizing Alzheimer’s disease (AD), and to reduce its secretion in numerous cell lines [12]. It was also suggested that RSV reduces neurodegeneration in the hippocampus and prevents learning deficits [15].

Currently, the use of RSV in humans remains limited due to its photosensitivity, easy oxidation [16], and low biodisponibility [1]. In addition, pharmacokinetic evaluations of free RSV have reported a very short half-life (30–45 min) and a rapid metabolism in rats [17]. Therefore, large doses of this polyphenol would be necessary to be effective in humans, which is limited by its low water solubility [18]. In order to overcome those limits, RSV has been encapsulated in order to favor its biological activity and increase its half-life [18]. Thus, many encapsulation methods have been used for this purpose, such as microemulsion [19,20], liposomes [21,22,23,24], and biopolymers [25,26]. Recent studies have focused on the association of RSV to nanoparticles. Indeed, Wang et al. modified liposomes encapsulating RSV with iron oxide nanoparticles for targeted PD treatment application [27]. RSV was also conjugated to silver and gold nanoparticles in order to enhance their antibacterial efficacy [28,29], or was used as an anticancer delivery system [30]. To the best of our knowledge, RSV has never been linked covalently to iron oxide nanoparticles. Therefore, the latter strategy has been chosen and herein we aim at delivering a RSV derivative, the molecules of which were grafted to the surface of superparamagnetic iron oxide nanoparticles (SPIONs), for further studies in neurodegenerative disease or glioma treatment.

SPIONs are considered as one of the most developed nanoparticles for various biomedical applications thanks to their small size, high magnetic moment, high surface to volume ratio, and biocompatibility [31]. They have been used for magnetic resonance imaging (MRI) as a contrast agent [32,33], cell labeling, tissue repair, gene and drug delivery, hyperthermia [34], and nano-sensors [35]. One of the most promising biomedical applications of SPIONs is drug delivery, thanks to the magnetic response of the iron oxide allowing magnetic targeting, which makes the retention of nanoparticles in the target tissue longer [36]. The most challenging targets for SPIONs are in the brain because of the presence of the BBB, a natural boundary between the neural tissue and the blood circulation, which limits the entrance of most drugs intended for the central nervous system (CNS). Many kinds of nanoparticles, polymeric and inorganic, have been studied for drug delivery across the BBB, however, the advantage of using inorganic nanoparticles (silica, gold, SPIONs, etc.) is the facility in modulating them in terms of shape, size, and surface modification [37]. The surface modification of nanoparticles increases their ability to cross this barrier. Indeed, unlike bare SPIONs, it has been reported that ligand-coated SPIONs or BBB disruption (BBB-targeting peptides, curcumin, polyethylene glycol (PEG)/chitosan, etc.) make them capable of crossing the BBB because they facilitate SPIONs uptake by the endothelial cells via specific receptors [38]. Moreover, the magnetic targeting of SPIONs not only enhances their penetration in brain cells, as has been proved by Chertok et al. on glioma cells [39,40], but also can transiently increase the BBB permeability following from magnetic heating (hyperthermia), as was proved by Tabatabaei et al. for rat brains [41]. The percentage of SPIONs that reach the CNS via the bloodstream has varied from 17 to 30% according to rodent studies [42].

Herein, we studied the surface modification of SPIONs with a derivative molecule of RSV, used as a therapeutic molecule, for further application in neurodegenerative diseases. This molecule has the same stilbene core as RSV—4’-hydroxy-4-(3-aminopropoxy) trans-stilbene, hereafter referred to as HAPtS (Figure 1)—and it was exclusively synthesized for this purpose. The anti-tumor and anti-microbial activities of compounds structurally close to HAPtS have been proven in previous studies [5,43]. The feature of this phenolic stilbene is an organic alkyl chain with a terminal functional group (primary amine) capable of linking to SPION’s surface, as shown in Figure 1.

## 2. Materials and Methods

### 2.1. Chemicals and Reagents

Iron (III) chloride hexahydrate (FeCl_3_.6H_2_O, 97%), and iron (II) chloride tetrahydrate (FeCl_2_.4H_2_O, 98%) were purchased from Alfa Aesar (Haverhill, MA, USA). Sodium hydroxide (NaOH, ≥97%), ammonium hydroxide solution (NH_4_OH, 28%), hydrochloric acid (HCl, 37%), nitric acid (HNO_3_, 69%), 3-chloropropyltriethoxysilane (CPTES, 95%), dimethylsulfoxide (DMSO, ≥99.7%), crystal violet, 2,2’-azobis (2-amidinopropane) dihydrochloride (AAPH), fluorescein sodium salt, *N,N*-diisopropylethylamine, ReagentPlus® (DIEA, ≥99%), 3-(4,5-dimethylthiazol-2-yl)-2,5-diphenyl tetrazolium bromide (MTT, 98%), 7β-hydroxycholesterol (7β-OHC), and Trolox (97%) were purchased from Sigma-Aldrich (St. Louis, MO, USA). Absolute ethanol (EtOH, ≥99.8%) was purchased from Fluka (Seelze, Germany). Phosphate buffer saline (PBS) 1× solution was purchased from Fisher Chemicals (Fair Lown, NJ, USA). Dulbecco’s modified Eagle medium (DMEM), trypan blue, fetal bovine serum (FBS), and antibiotic (Penicillin, Streptomycin) were purchased from Dominique Dutscher and Pan Biotech (Brumath, France). Ultrafiltration membranes (regenerated cellulose 100 kDa) were acquired from Merck Millipore (Darmstadt, Germany).

### 2.2. Characterization Techniques 

An X-Ray diffraction (XRD) pattern of bare SPIONs was obtained using a Bruker D8 Advance diffractometer (Billerica, MA, USA). Cu Kα_1,2_ radiations (λα_1_ = 1.540598 Å and λα_2_ = 1.544426 Å) were applied. Scans were measured over a 2θ range of 10–80°. A step of 0.026° and a scan speed of 52 s per angle unit were set. The data analysis was carried out with Topas® software (Billerica, MA, USA). The Le Bail method was used to obtain lattice parameters and mean crystallite size. 

The morphology of synthesized nanoparticles (NPs) were observed by Transmission electron microscopy (TEM, Tokyo, Japan) using a JEOL JEM 2100F microscope operating at 200 kV (point-to-point resolution of 0.19 nm). The samples were prepared by evaporating a diluted suspension of SPIONs in deionized (DI) water on a carbon-coated copper grid. About 150 nanoparticles were counted in order to estimate their average size (imageJ software, 1.52a, NIH, MD, USA).

X-ray photoelectron spectroscopy (XPS) measurements were collected with a PHI 5000 Versaprobe instrument (ULVAC-PHI, Osaka, Japan) using an Al Kα monochromatic radiation (EKα(Al) = 1486.7 eV with a 200 μm diameter spot size)). Powders were pressed on an indium sheet. Data were analyzed with CasaXPS processing and ULVAC-PHI MultiPak software (ver. 9.0.1, Osaka, Japan) for quantitative analysis. Neutralization was used to minimize charge effects. C1s peak at 284.5 eV was used as reference. Shirley background was subtracted, and Gauss (70%)–Lorentz (30%) profiles were used. Full width at half maximum (FWHM) were fixed between 1.4 and 1.6 eV for O 1s, 1.6–1.7 eV for C 1s, and 1.7−1.8 eV for N 1s.

Specific surface area measurement (S_BET_) was carried out using a Micromeritics Tristar II apparatus. Samples were outgassed in situ at 100 °C under a pressure of *ca*.≈30 μbar. The measurements were performed at liquid N_2_ temperature using N_2_ as the adsorbing gas. The BET method was used in the calculation of surface area value from the isotherm of nitrogen adsorption. The mean apparent particle diameter was determined from surface area. 

Zeta potentials were measured with a Malvern ZetasizerNano ZSP instrument (Worcestershire, UK) supplied by a DTS Nano V7.11 software (Worcestershire, UK). The suspensions of SPIONs were prepared in 10^−2^ M NaCl aqueous solutions. The pH of the suspension was adjusted from 3 to 11 by addition of HCl (0.1 M) or NaOH (0.1 M and 0.01 M) solutions. Hydrodynamic diameters were determined by Dynamic Light Scattering (DLS, Malvern, Worcestershire, UK) curves, which are derived from the number distribution calculation on the same instrument. Measurements were carried out three times at 25.0 °C, immediately after homogenization by ultrasound bath for 5 min, and using refractive index 2.42 for Fe_3_O_4_ and 1.33 for water (viscosity 0.8872 cP).

The weight losses of bare and grafted SPIONs were studied by thermogravimetric analyses (TGA) with a TA Instruments Discovery TGA (Newcastle, UK) under an air flow rate of 25 mL min^−1^. The analyses were done using the following thermal program: ramp 1 of 20 °C.min^−1^ from 25 °C to 100 °C, isotherm at 100 °C for 30 min (to remove the remaining moisture), and ramp 2 of 5 °C min^−1^ from 100 °C to 700 °C. TGA weight losses were considered only from the second ramps.

Fourier transform infrared (FTIR) measurements were recorded on a Bruker IFS 28 (Billerica, MA, USA) using OPUS version 3.1 in the wavenumber range of 4000–400 cm^−1^, with a resolution of 4 cm^–1^ and a total of 50 scans per measurement. Pellets were made of 0.5 mg sample mixed in 199.5 mg of dried KBr.

UV-visible spectroscopy (UV-vis) measurements were carried out using a Shimadzu UV-2550 UV-Vis spectrophotometer (Tokyo, Japan). All spectra were measured in the range of 220-800 nm and recorded at 23 °C using UV cuvettes of 1 cm path length. 

### 2.3. Synthesis of 4’-Hydroxy-4-(3-aminopropoxy) Trans-Stilbene (HAPtS) Molecule

The 4’-hydroxy-4-(3-aminopropoxy)*trans*-stilbene (HAPtS) was synthesized by a Wittig reaction from 4-acetoxybenzyltriphenylphosphonium and N-3-(4-carbaldehydephenoxy)propylphtalimide. The Wittig reaction was carried out in phase transfer conditions [44] to give 4-acetoxy-4’-N-(3-O-propylphtalimide) *trans*-stilbene. The protective groups of latter molecule were removed to afford HAPtS. A detailed description, scheme, and NMR data of each reaction step of HAPtS synthesis are given in the Supplementary Materials (SM1).

### 2.4. Synthesis of Bare and Modified Nanoparticles

#### 2.4.1. Synthesis of Bare SPIONs

Bare superparamagnetic iron oxide nanoparticles (SPIONs) were synthesized following a simple co-precipitation protocol. Briefly, a stoichiometric mixture of FeCl_2_·4H_2_O (12.72 g) solution and FeCl_3_·6H_2_O (34.58 g) solution was prepared in 1.5 L of deionized (DI) water in the molar ratio Fe^2+^:Fe^3+^ = 1:2 at 25 °C. After the total dissolution of salts, 120 mL of ammonium hydroxide (28%) was added quickly to the solution and the SPIONs were precipitated immediately. The solution was kept under magnetic stirring for 2–3 minutes and then washed thoroughly with deionized (DI) water, using a magnetic separation, until the solution reached pH 8. The pH of the suspension was adjusted to pH 3 with HNO_3_ solution (0.1 M). The obtained suspension was dialyzed for 76 h against pH 3 HNO_3_ solution. After that, the suspension was centrifuged at 20,000 *g* for 15 min and only the supernatant was kept as a homogeneous and stable suspension. The final SPION concentration of 9.2 mg·mL^−1^ of nanoparticles was used for further surface modifications. A small amount of the obtained suspension was freeze-dried for characterization. 

#### 2.4.2. Synthesis of 3-Chlorporyltriethoxysilane-Modified SPIONs: SPIONs-CPTES

A total of 50 mg of as prepared SPIONs were dispersed in a mixture of EtOH and DI water, with a volume ratio 3:1. 600 µL of CPTES, which was added to the suspension and the pH was then adjusted to 4 with NaOH solution (0.1 M). The suspension was kept under magnetic stirring at 360 rpm for 24 h at 25 °C.

The purification of the grafted SPIONs from the unreacted CPTES was realized using an ultrafiltration device through an ultrafiltration membrane (regenerated cellulose, 30 kDa). The purification was carried on until the conductivity of the filtrate reached that of DI water (0.5 µS·cm^−1^). 

#### 2.4.3. Synthesis of 4’-hydroxy-4-(3-aminopropoxy)-trans-stilbene-Modified SPIONs: SPIONs-CPTES-HAPtS

A total of 7.5 mg of CPTES-modified SPIONs (SPIONs-CPTES) were dispersed in anhydrous DMSO and 80 µL of DIEA (organic base), and 1.9 mg of HAPtS was added in excess to the SPIONs suspension (molar ratio HAPtS/grafted CPTES is 7:0.4). The suspension was kept under magnetic stirring (360 rpm) for 24 h at 25 °C. The purification was carried out by a magnetic decantation with DMSO and then DI water. The purification efficiency was controlled by UV-visible spectrometry (of the washing water).

**Figure 2:**
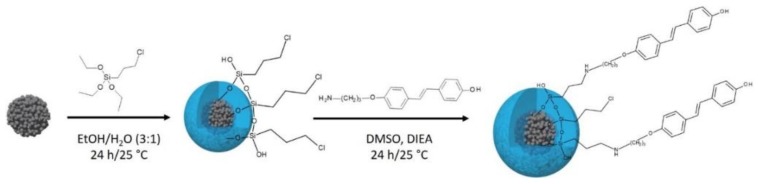
Schematic representation of the *trans*-resveratol derivative-modified SPIONs (SPIONs-CPTES-HAPtS) nanohybrid synthesis.

After each synthesis step, a small amount of the SPIONs in suspension was freeze dried for 48 h to perform the suitable characterizations, and the rest of the samples were kept in suspension to avoid nanoparticles agglomeration during the drying step and to keep a good dispersion of SPIONs in water. Figure 2 summarizes the surface modification steps of bare SPIONs.

### 2.5. Biological Assays

#### 2.5.1. Antioxidant Test: the Oxygen Radical Absorbance Capacity (ORAC) Assay

The antioxidant capacity of HAPtS was evaluated by the ORAC test, which measures the ability of a molecule to prevent or delay the oxidation of a fluorescent molecule (in this case fluorescein) in the presence of a free radical-generating (oxidizing) molecule, which is AAPH. The experiment was carried out according to the literature [6,45]. Briefly, the reaction was carried in a 96-well black plate, in 75 mM phosphate buffer (pH 7.4), and the final reaction mixture was 200 µL; 20µL of antioxidant (Trolox for calibration curves, HAPtS or RSV) molecules and 120 µL of fluorescein solution (final concentration of 50 nM) were added in the 96 wells of the microplate. The mixtures were incubated for 15 min at 37 °C, and 20 µL of AAPH solution (40 mM, final concentration) was added rapidly using the automate (Tecan machine). The fluorescence was recorded every minute for 80 min. The microplate was automatically shaken prior each reading. 

The calibration curve was carried out using five calibration solutions of Trolox (1–50 µM, final concentration) and a blank of only fluorescein and AAPH was also used. All the reaction mixtures were prepared in triplicate. The obtained curves (fluorescence versus time) were normalized and the ORAC values (AUC) were calculated from the area under the fluorescence decay curve according to the following formula:

$$\mathrm{AUC}=1+\sum_{i=1}^{i=80}f_{i}/f_{0}$$

where f_0_: fluorescence read at 0 min and f_i_: fluorescence read at i min.

The relative AUC values (expressed in equivalent Trolox) are determined as following:

$${\mathrm{AUC}}_{\mathrm{relative}}=\frac{{\left(\mathrm{AUC}\right)}_{\mathrm{sample}}}{{\left(\mathrm{AUC}\right)}_{\mathrm{Trolox}}}\times \frac{\left[\mathrm{Trolox}\right]}{\left[\mathrm{sample}\right]}$$

#### 2.5.2. Cell Culture 

The C6 rat glioma cells were cultured at 25,000 cells.cm^−2^, in 6-well or 96-well plates, in Dulbecco’s modified Eagle medium (DMEM) with 10% heat inactivated fetal bovine serum FBS and 1% antibiotic (Penicillin, Streptomycin), as described by Nury et al. [46]. The cells were incubated at 37 °C in a humidified atmosphere containing 5% CO_2_ and passaged twice a week. At each passage, cells were trypsinized with a 0.05% trypsin–0.02% ethylenediaminetetraacetic acid (EDTA) solution (Pan Biotech).

#### 2.5.3. Clonogenic Survival Assay

Cell clonogenic survival assay is an in vitro cell survival assay based on the ability of a single cell to grow into a colony following insult with physical or chemical agents [47]. After cell incubation for 24 h with a density of 25,000 cells cm^−2^, cells were exposed to the following compounds: RSV (50 µM), HAPtS (50 µM), grafted SPION (SPIONs-CPTES-HAPtS (determined volume of nanohybrids suspension which corresponds to 50 µM of grafted HAPtS)), SPIONs-CPTES, bare SPIONs, positive control (cells treated with a toxic molecule, 7β-hydroxycholesterol (7β-OHC), 100 µM). Data were compared to untreated cells. It is important to note that the masse of SPIONs-CPTES sample corresponds to that included in the sample SPIONs-CPTES-HAPtS and it is the same for bare SPIONs. At the end of treatment in 6-well plates, cells were trypsinized, counted on a Malassez hemocytometer using trypan blue dye (v:v), and 1000 cells were cultured in a 100 mm diameter Petri dish containing 10 mL of culture medium for 8 days. After 8 days of culture, the cells were stained with crystal violet solution to visualize the cell colonies (the culture medium was changed twice a week).

#### 2.5.4. Cytotoxicity: MTT Assay

MTT (3-(4-,5-dimethylthiazol-2-yl)-2,5-diphenyltetrazolium bromide) assay was used to evaluate the effects of molecules and nanoparticles on cell viability and was carried out as described by Lizard et al. [48]. Cell viability was assessed as a function of the mitochondrial activity. Cells were seeded in 96-well plates at an initial seeding density of 25,000 cells·cm^−^2, and incubated for 24 h at 37 °C, 5% CO_2_. After exposure to RSV (0.5–50 µM), HAPtS (0.5–50 µM), bare SPIONs (50–500 µg·mL^−1^ of SPIONs), SPIONs-CPTES (50-500 µg·mL^−1^ of SPIONs), SPIONs-CPTES- HAPtS (50–500 µg·mL^−1^ of SPIONs), and 7β-OHC (100 µM) for 24 h, cells were incubated with MTT (0.05 mg·mL^−1^) for 3 h in the dark. The formazan crystals formed were solubilized using 200 µL DMSO and absorbance was read at 570 nm with a microplate reader (Tecan, France).

#### 2.5.5. Cytotoxicity: FDA (Fluorescein Diacetate) Assay

The FDA assay stains cells fluorescently green when they have intact cell membrane esterase activity. All non-FDA-fluorescent cells are considered dead [49]. Cells were seeded in 96-well plates at an initial seeding density of 25,000 cells/cm^−^2, and incubated for 24 h at 37 °C, 5% CO_2_. After exposure to RSV (0.5–50 µM), HAPtS (0.5–50 µM), bare SPIONs (50–500 µg·mL^−1^ of SPIONs), SPIONs-CPTES (50–500 µg·mL^−1^ of SPIONs), SPIONs-CPTES- HAPtS (50–500 µg·mL^−1^ of SPIONs), and 7β-OHC (100 µM) for 24 h, cells were washed twice with 200 µL PBS and then treated with 150 µL of FDA solution (50 µM) for 5 min at 37 °C. FDA solution was then removed and 150 µL of lysis buffer (aqueous solution with 10% (v/v) SDS and 0.079% (m/v) Tris HCl) was added. The 96-well plate was kept under magnetic stirring for 3 min and the fluorescence was read (λ_exc_ = 485 nm, λ_em_ = 528 nm) with a microplate reader (Tecan).

## 3. Results and Discussion

### 3.1. Characterization of the New RSV Derivative Molecule: HAPtS

**Figure 3:**
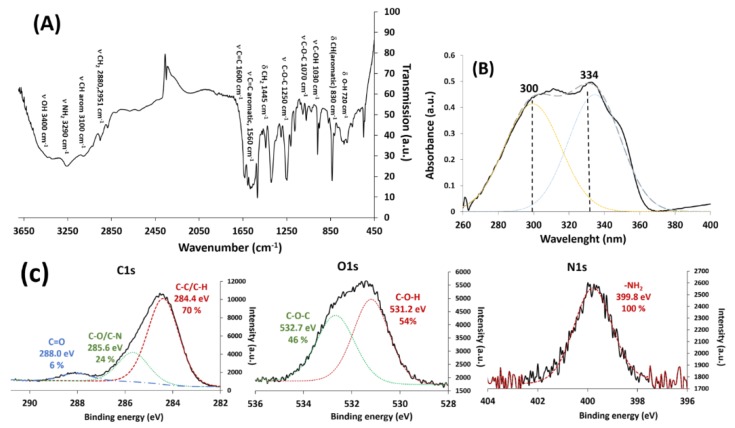
(**A**) Fourier-transform infrared (FTIR) spectrum, (**B**) UV-visible spectrum, and (**C**) fitted X-ray photoelectron spectroscopy (XPS) spectra of C1s, O1s, and N1s peaks of the synthesized RSV derivative molecule (HAPtS).

Figure 3A shows the FTIR spectrum of the as-synthesized RSV derivative molecule. It proves the presence of most of the characteristic bands of the stilbene derivative—the stretching vibration bands of NH_2_ and OH groups are situated at 3400 and 3290 cm^−1^, respectively, and those of -CH_2_ (νCH_2_) are at 2951 and 2880 cm^−1^. The stretching vibration band of ν C-O-C, ν C-O-H, and ν C-N bonds is illustrated by an intense wide band at 1250 cm^−1^, as well as by two less intense and finer bands around 1030 and 1070 cm^−1^. The vibration bands of C=C bonds in the aromatic and inter-aromatic rings are observed at 1560 cm^−1^ and 1600 cm^−1^, respectively. Moreover, the bending vibration band of C-H in aromatic rings (δ C-H) is observed at 830 cm^−1^ [50]. All these bands are in common with those observed in the commercial RSV molecule and are consistent with the data in literature [51].

The fitted UV-Visible spectrum (fityk software) of HAPtS in DMSO, shown in Figure 3B, proves the presence of a broad band with two contributions centered at 300 and 334 nm, which are characteristic of the trans isomer of resveratrol molecule [52]. The second absorbance peak at 334 nm indicates the deprotonated form of resveratrol according to the study by Manuel et al. [53]. This form of stilbene derivative is justified by the use of potassium hydroxide (KOH) during the synthesis and the extraction process of HAPtS molecules (see Supplementary Materials SM1).

XPS analysis shows the experimental and the calculated elemental analysis of the synthesized stilbene derivative (Table 1). The presence of a small amount of silicon and calcium in the final product is due to the purification step, in which a chromatography on silica gel column was used. In order to better understand the type of chemical bonds in HAPtS molecule, a fitting of C1s, O1s, and N1s XPS spectra was carried out.

Figure 3C shows three components in C1s XPS peak, which can be assigned to three types of carbons: C=O, C-O/C-N, and C-C/C-H, situated, respectively, at 288.0, 285.6, and 284.4 eV binding energies. However, according to the chemical structure of HAPtS (Figure 1), only two types of carbon should exist, which are C-C/C-H and C-O/C-N. This suggests that the presence of carbonyl groups (-C=O) could be attributed to the presence of calcium carbonate compounds, as the calcium exists as an impurity in the final product, and since K_2_CO_3_ is used along the synthesis (see SM1). This result explains the difference in the C/N ratio between the calculated and the experimental structure Table 1. On the other hand, Table 2 shows a similarity between the calculated or theoretical and the experimental percentages of the carbon, oxygen, and nitrogen chemical bonds, obtained from the fitting of XPS spectra in Figure 3C. Additionally, NMR data show the presence of the characteristic peak of HAPtS (SM1).

FTIR, UV-vis XPS, and NMR results suggest that the molecule synthesized is the sought stilbene derivative molecule—HAPtS.

**Figure 4:**
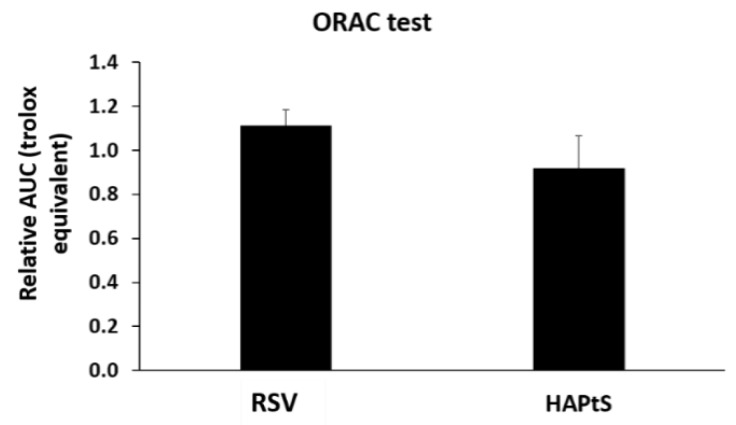
Antioxidant capacity measurement of *trans*-resveratrol (RSV) and chemically synthesized molecules (HAPtS).

Figure 4 gives the antioxidant capacity of RSV and HAPtS molecules, estimated by ORAC test and expressed in Trolox equivalent. The result indicates that the HAPtS has almost the same ORAC value as the RSV molecule, with 1.1 ± 0.1 and 0.9 ± 0.2 Trolox equivalent, respectively. This proves that the antioxidant capacity of HAPtS remains despite the chemical change and the presence of an alkyl chain.

### 3.2. Elaboration of HAPtS Nanocarrier Based on Superparamagnetic Iron Oxide Nanoparticles (SPIONs)

The crystallinity of the synthesized powder of bare SPIONs was checked by the XRD pattern, shown in Figure S1 (in Supplementary Materials), which corresponds to the spinel crystallographic phase of either magnetite (Fe_3_O_4_) or maghemite (γ-Fe_2_O_3_) with a crystallite size of 10.1 ± 0.1 nm. The lattice parameter, determined from XRD pattern, is 8.368 ± 0.001 Å and the diffracting planes are (220), (311), (400), (511), and (440). The interatomic distances observed on the selected area diffraction pattern of Fe_3_O_4_ are in agreement with the spinel structure (ICDD: 19-0629) [54]. The lattice parameter suggests that the powder is slightly oxidized (Fe_3(1−__δ)_O_4_ crystallites, with δ the deviation from oxygen stoichiometry and a small presence of γ-Fe_2_O_3_ on the surface) [55,56].

**Figure 5:**
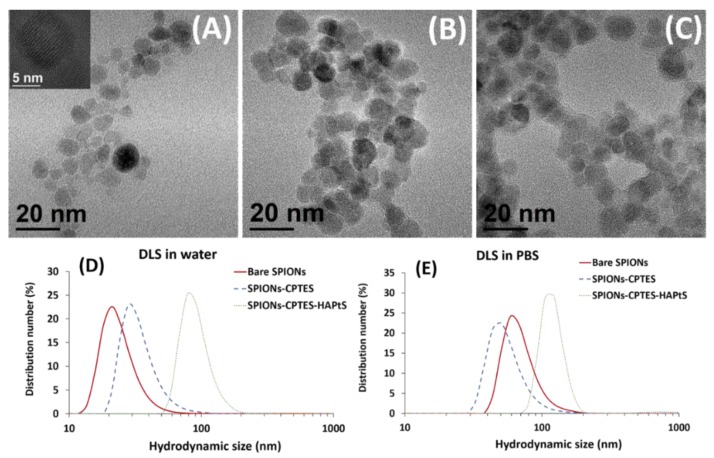
Transmission electron microscopy (TEM) images of (**A**) bare superparamagnetic iron nanoparticles (SPIONs) (inset: high resolution (HR)-TEM image of one nanoparticle), (**B**) SPIONs-CPTES, and (**C**) SPIONs-CPTES-HAPtS and dynamic light scattering (DLS) curves of bare and functionalized SPIONs in (**D**) water (10^-2^ NaCl) and (**E**) phosphate buffer solution (PBS) 0.1× (pH 7.4).

The specific surface area (S_BET_) of bare SPIONs is 114.0 ± 0.5 m^2^·g^−1^. The calculated crystallite size from S_BET_ value is 10.1 ± 0.2 nm, which is in a good agreement with the XRD result. Moreover, the counting of hundreds of SPION nanoparticles from TEM images (Figure 5) gives an average size of 9.0 ± 2.0 nm, illustrating the two previous methods of crystallite size measurement (S_BET_ and XRD). 

Figure 5 shows the evolution in the size of bare and functionalized SPIONs, assessed by dynamic light scattering (DLS) and TEM images. Unlike the latter that do not show a significant increase in the size of SPIONs after grafting, DLS measurements prove considerable changes in the hydrodynamic size (d_H_) of the functionalized SPIONs. Indeed, the statistic measurement of nanoparticle size using TEM images shows an average size of about 9 nm for bare and grafted SPIONs (Table 3), however, Figure 5B,C suggests also the presence of an organic shell around the iron oxide inorganic core. The organic shell is considered for size measurements by DLS, but it cannot be the main reason for the increase of the d_H_. Indeed, the particle size as measured by DLS is generally higher than the size of nanoparticles observed via TEM because it takes into account not only the hydration layer surrounding the nanoparticles but also the aggregated nanoparticles. This explains the size of bare SPIONs of 21 ± 8 nm and the increase in the hydrodynamic size after CPTES and HAPtS grafting to 26 ± 7 nm and 92 ± 20 nm, respectively (Figure 5D, Table 3).

**Figure 6:**
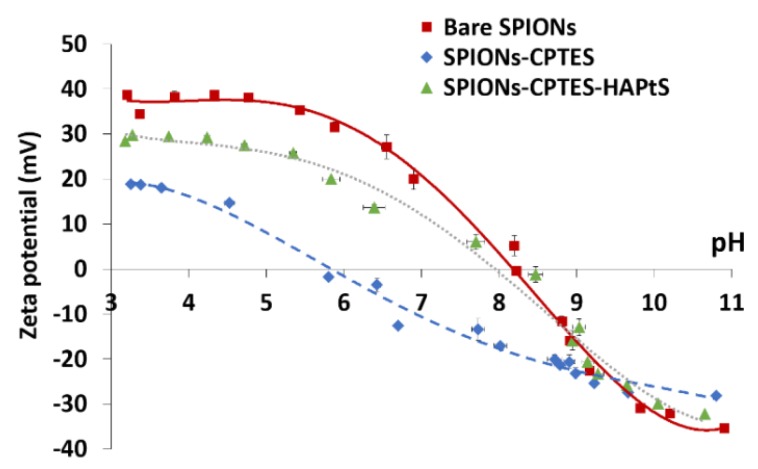
Zeta potential measurements of bare and functionalized SPIONs (10^-2^ M NaCl).

On the other side, the hydrodynamic size of bare SPIONs in water (pH 5) is different from that measured in PBS medium (60 ± 9 nm), since the pH of the latter is close to the isoelectric point (IEP) of bare SPIONs (Figure 6), which makes nanoparticles aggregate rapidly. The aggregation of bare SPIONs in PBS medium is also proved by the increase of the polydispersion index (PDI) from 0.157 to 0.489.

Figure 5E suggests that SPIONs-CPTES is the most stable sample in PBS medium, with the smallest hydrodynamic size (50 ± 8 nm), compared to SPIONs-CPTES- HAPtS sample (118 ± 25 nm). Moreover, Figure S2 in the Supplementary Information (SI) illustrates the improvement in the colloidal stability between the bare and functionalized SPIONs in PBS medium (pH 7.4) after 24 h. In fact, functionalized SPIONs remain in suspension with good colloidal stability compared to the poor stability of bare SPIONs, which completely settled. Nanoparticle aggregation of bare SPIONs are not observed in Figure 5E, since DLS measurements were carried out immediately after homogenization. We can conclude from the previous results that the increase of the hydrodynamic size in water (pH 5, 10^−2^ M NaCl) after the sequential grafting is explained by the increase of the hydration layer around the nanoparticles, but also due to nanoparticle aggregation, explained by the increase of interaction between nanohybrids. This interaction seems to increase after the grafting of the hydrophobic molecule, which is HAPtS through the pi-stacking bonds between the aromatic rings that could be intra and inter-nanoparticles during or after the surface modification of SPIONs. On the other side, when the medium changes, in particular the pH (7.4) and the ionic strength (PBS, 0.1×), the dH of all samples increases continuously, compared to dH in water. We notice also that the negatively charge nanoparticles as SPIONs-CPTES (Figure 6) have better colloidal stability in PBS medium than the positively charged samples (Bare SPIONs and SPIONs-CPTES-HAPtS). This result shows that the colloidal stability of the samples is also governed by the electrostatic interactions. On one side, the Debye length decreases with the increasing ionic strength, which favors the decrease of the particle repulsion, and thus the agglomeration. On the other side, when the pH of the medium increases, it becomes close to IEP of bare and SPIONs-CPTES-HAPtS, which explains also the increase of dH of these two samples. However, the aggregation phenomenon in SPIONs-CPTES-HAPtS is reduced compared to bare SPIONs due to the presence of two organic layers (CPTES and HAPtS). These two molecules bring steric repulsion that limit the interactions to pH 7.4. Otherwise, these results suggest that the surface modification of nanoparticles participate to stabilize the nanoparticles.

In order to better understand the evolution of the colloidal stability of the synthesized nanohybrids in biological medium, the hydrodynamic size of all samples was followed up in PBS medium (0.1×, pH7.4), with addition of bovine serum albumin as a function of the time at 37 °C. The results presented in Figure S3 show that bare SPIONs and SPIONs-CPTES-HAPtS have almost a constant dH during the time, approximately 125 nm and 65 nm, respectively, however, dH of SPIONs-CPTES decreases from 290 nm to about 200 nm. We notice that contrarily to PBS medium only, the addition of BSA has contributed to stabilizing bare SPIONs at physiological pH and decreases the dH of bare and HAPtS-modified SPIONs. However, BSA addition increases the dH of CPTES-modified SPIONs compared to PBS only. This result shows that the dispersion medium composition is very important in the control of the colloidal stability of nanoparticles, which was also proved in our previous work for titanate nanotubes [57]. The poor stability of SPIONs-CPTES sample can be explained by the interaction between BSA and the latter sample due to the presence of proteins containing free amine groups, which could interact with the free chloride in CPTES molecule.

Zeta potential measurements provide information about the surface modification of SPIONs following a molecule grafting, as well as about their colloidal stability. Figure 6 shows the evolution in the zeta potential curves as a function of pH for the synthesized nanohybrids. It illustrates a decrease in the zeta potential from about 40 mV to 20 mV, at the lowest imposed pH of about 3, and in the isoelectric point (IEP) from 8.2 to 5.9 after CPTES grafting. Although the organic chain in CPTES should not change the IEP of grafted SPIONs because it is considered as a neutral organofunctional silane, the decrease in the global surface charge may be due to the presence of silanol groups (Si-OH) on SPIONs surface, as is the case in silica nanoparticles, which exhibit a low IEP (below pH 3) [58]. After HAPtS grafting, the zeta potential as well as the IEP increase to about +30 and 8.3 mV, respectively, at the lowest imposed pH of about 3. Such a considerable shift is due to the presence of protonated amine group (pk_a_ between 9 and 10) in the trans-resveratrol derivative molecule, which leads to a positive surface charge on SPIONs’ surface [58].

**Figure 7:**
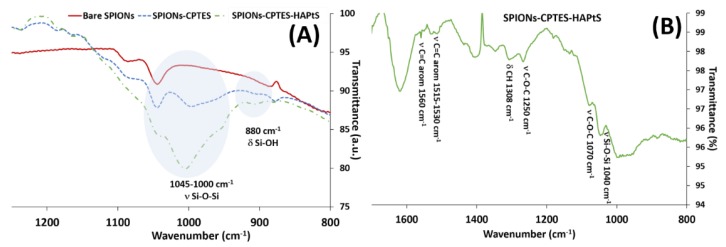
Infrared spectra of (**A**) bare SPIONs, SPIONs-CPTES, and SPIONs-CPTES-HAPtS (spectra range from 1250 to 800 cm^−1^), and (**B**) SPIONs-CPTES-HAPtS (from 1700 to 800 cm^−1^).

The synthesized nanohybrids were analyzed by infrared spectroscopy, where only the interesting part of the spectra are shown in Figure 7A, while the whole spectra are shown in Figure S4. The IR spectra illustrate the grafting of CPTES by the presence of its characteristic vibration bands, which are mainly the broad stretching vibration band of siloxane bonds (ν Si-O-Si) in the range between 1045 and 1000 cm^−1^ and the bending vibration band of Si-OH at 880 cm^−1^ [50,59]. Indeed, the vibration of Si-O-Si bond formation is due to the hydrolysis condensation mechanism of oligosiloxane containing Si-OH bonds [60]. In addition, the immobilization of HAPtS on SPION’s surface is illustrated in Figure 7B by the presence of the stretching vibration band of the aromatic rings (ν C=C at 1560 and 1515 cm^−1^ and δ CH at 1308 cm^−1^) and C-O bonds (ν C-O at 1250 and 1070 cm^−1^). The stretching vibration bands of the alkyl chain (-CH_2_-CH_2_-) are also observed at 2960-2930 cm^−1^.

The elemental analysis by XPS shows the evolution in the surface chemistry of SPIONs depending on the kind of the grafted molecule (Table 4). Indeed, the CPTES grafting is proved by the appearance of new elements, which are silicon (5.3%) and chlorine (2.2%), besides the increase of the carbon proportion from 3% for bare SPIONs to 15% for SPIONs-CPTES sample. The amount of carbon continues to increase following the surface modification of SPIONs-CPTES with HAPtS to 21.6%. However, the decrease in Cl/Si ratio from 0.4 to 0.2 after HAPtS immobilization proves the reaction between CPTES and the stilbene derivative through a nucleophile substitution occurred between nitrogen and chlorine. This result corroborates the re-appearance of nitrogen in the SPIONs-CPTES- HAPtS sample (Table 4). On the other hand, the atomic percentages of iron and silicon decrease continuously after each grafting step, which can be explained by a partial hiding of the inorganic core by the organic shell on the surface.

**Figure 8:**
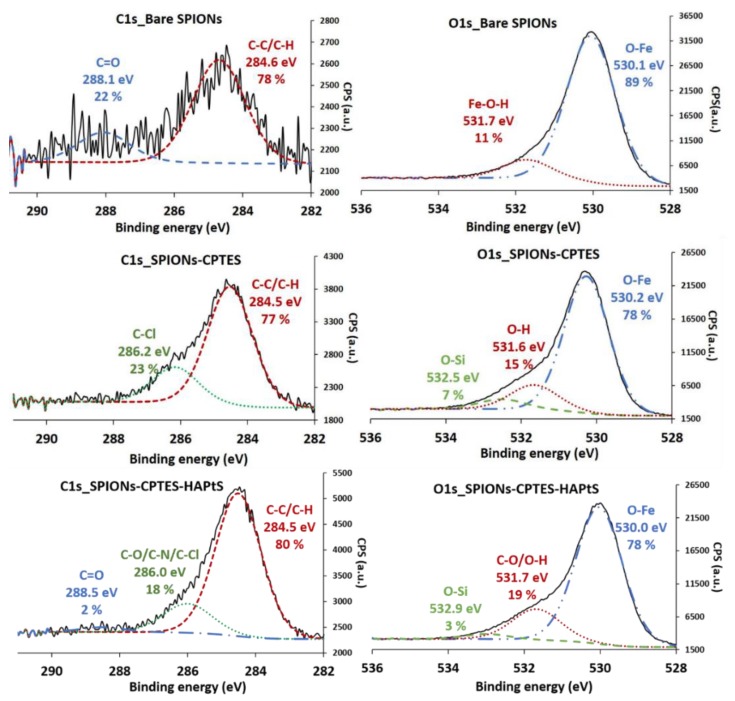
XPS spectra of C1s and O1s of bare SPIONs, SPIONs-CPTES, and SPIONs-CPTES-HAPtS samples.

In order to better investigate the change occurring in the chemical bonds on SPIONs surface, C1s and O1s XPS peaks are fitted, as is shown in Figure 8. It should be noted that the carbon present in bare SPIONs comes from the adventitious carbon contamination. C1s XPS peak of SPIONs-CPTES shows two components that correspond to two types of carbon bonds in this sample, which are C-C/C-H at 284.5 eV (77%), assigned to the organic alkyl chain, and C-Cl bond at 286.2 eV (23%). The high binding energy of C-Cl component can be explained by the high electronegativity of chlorine compared to hydrogen. It should be noted that it is not possible, in our case, to see the Si-C bond that must be overlapped by C-C/C-H peak. However, the experimental ratio of the percentage of Si-C bond (23%) regarding the total carbon bond is similar to the theoretical one (25%) (Figure 8). The C1s XPS peak for SPIONs-CPTES-HAPtS shows a decrease in the ratio between both contributions located at 286.0 eV and 284.5 eV (18%/80%), compared to that obtained for SPIONs-CPTES sample (23%/77%), which can be explained by the high amount of C-C/C-H bonds in HAPtS molecules.

The oxygen O1s XPS peak of bare SPIONs (Figure 8) shows the presence of two components—one is located at 530.1 eV and is attributed to the oxygen of the inorganic core (Fe-O-Fe), and the second at 531.7 eV corresponds to the surface oxygen bond Fe-OH [54]. After the first grafting step of the organosilane, a third component appears at high binding energy (532.5 eV) in the O1s peak of the SPIONs-CPTES sample. This component could be assigned to the Si-O-Si bonds of the oligosiloxane generated onto SPIONs surface. Moreover, the increase in O-H bond from 11% to 15% (Figure 8) can be also explained by the presence of silanol bonds (Si-O-H) in addition to those of Fe-OH. These results agree with the previous infrared analysis (Figure 7A). The percentage of O-H contribution increases continuously after the HAPtS grafting (19%), since in addition to Si-OH and Fe-OH bonds, there are C-OH bonds belonging to the RSV derivative. On the other hand, there is a decrease in the amount of Si-O bonds in SPIONs-CPTES-HAPtS to 3% instead of 7% in SPIONs-CPTES due to the presence of a thick organic shell that hides the inorganic core. The existence of N1s peak for SPIONs-CPTES-HAPtS, shown in Figure S5, proves the presence of HAPtS in this sample at a position almost similar to that of free HAPtS molecules (399.6 eV). It should be noted that the nitrogen present in bare SPIONs comes from the washing process in which HNO_3_ solution is used. 

**Figure 9:**
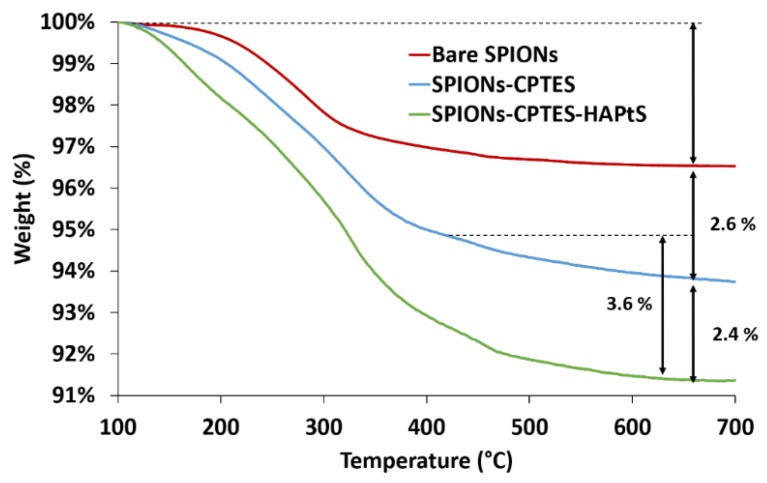
Thermogravimetric analysis (TGA) curves of bare and grafted SPIONs under air (100–700 °C).

The quantification of the immobilized trans-resveratrol derivatives (HAPtS) on SPIONs’ surface is carried out using thermogravimetric and XPS analyses. Indeed, the TGA curve of bare SPIONs reveals the weight loss of physisorbed water at low temperatures (100–200 °C) and chemisorbed water at high temperatures (200–700 °C) (Figure 9). The weight loss of functionalized SPIONs comes not only from physi- and chemisorbed waters, but also from the loss of the organic layers on SPIONs’ surface. This is also observed through the derivative curves of TG analysis (DTG), presented in Figure S6, which shows an increase in the DTG signal and the appearance of an additional peak after each grafting step. The grafting rate of SPIONs is expressed in molecule·nm^−^² of SPIONs or µmol of grafted molecule·g^−1^ SPIONs (the calculation details are given in Supplementary Materials (SM2)). The weight loss of CPTES corresponds to the difference between the bare SPIONs and the SPIONs-CPTES sample, which is 2.6%, and the grafting rate is estimated at 1.8 CPTES/nm² (Table 5). However, on the subsequent functionalization step, the estimation of the grafting rate of HAPtS becomes more difficult, since two different molecules are present on SPIONs’ surface (free CPTES and CPTES-HAPtS). Indeed, during the HAPtS grafting, 50% chlorine reacts with HAPtS, according to XPS data (Table 4), and since chlorine represents about 46% of the molecular weight of the degraded molecules during the heating process (M(Cl) = 35.5 g·mol^−1^ and M((-CH_2_-)_3_) = 42 g·mol^−1^), the real weight loss corresponding to the last grafting can be estimated at 3.6% (2.4 + (2.6 × 0.46)) instead of 2.4%.

### 3.3. Assessment of Nanohybrids Biological Efficiency

#### 3.3.1. Cytotoxicity Assessment

The nanoparticles’ cytotoxicity was studied by MTT and FDA tests in order to assess the effect of nanoparticles on the viability of C6 rat glioma cells. Indeed, the FDA test is a test for estimation of viable cells, where the fluorescein diacetate (FDA) passes through cell membranes and is then hydrolyzed by intracellular esterases (in living cells) to produce fluorescein, which is accumulated inside cells and exhibits green fluorescence [31]. This test assesses the cells viability through the cellular membrane injuries or damages. This test was carried out for RSV (50 µM), HAPtS (50 µM in RSV equivalent), bare, and grafted SPIONs (SPIONs-CPTES and SPIONs-CPTES-HAPtS) compared to the untreated cells (Ctrl), and the 7β-hydroxycholesterol (7β-OHC, 100 µM) was used as a positive control of toxicity.

**Figure 10:**
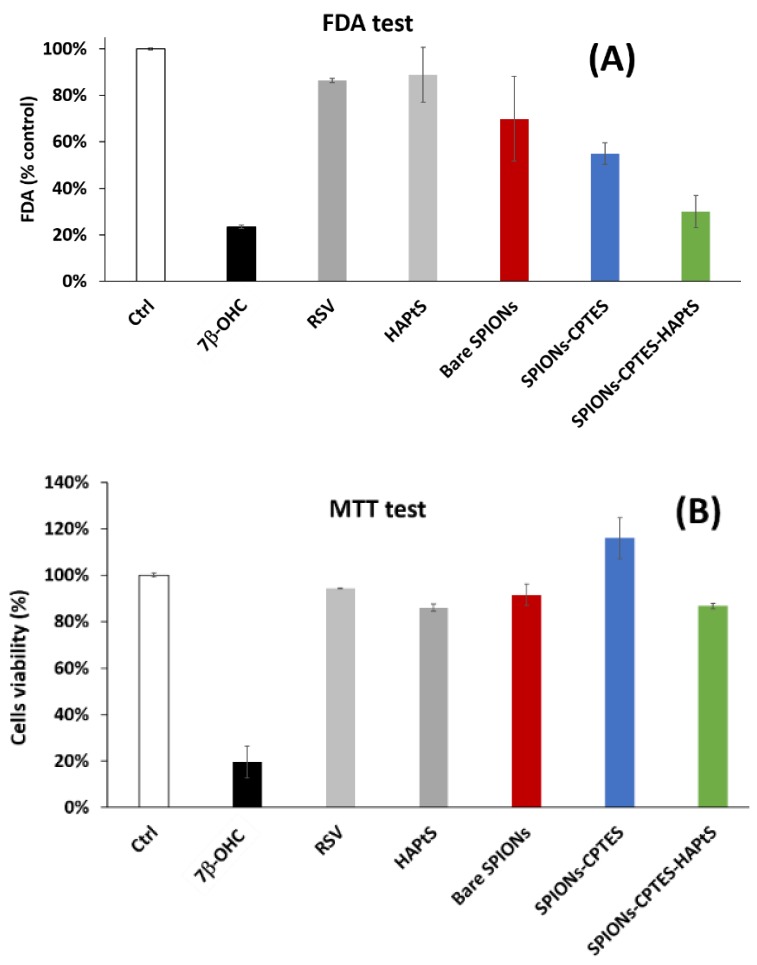
Cytotoxicity tests ((**A**) FDA and (**B**) MTT) for control (Ctrl; untreated cells), positive cytotoxic control (7β-OHC, 100 µM), RSV (50 µM), HAPtS (50 µM in RSV equivalent), bare SPIONs (the same amount as in SPIONs-CPTES-HAPtS), SPIONs-CPTES (the same amount as in SPIONs-CPTES-HAPtS), and SPIONs-CPTES-HAPtS (amount equivalent to 50 µM HAPtS). Incubation time of cells with molecules or nanoparticles was 24 h and cells are C6 rat glioma cells.

The results of the FDA test suggest that the surface modified SPIONs (SPIONs-CPTES and SPIONs-CPTES-HAPtS) have a different effect on the cells membrane. Indeed, Figure 10A shows that no significant damage in the cellular membrane is observed for bare SPIONs, free RSV, and HAPtS molecules. However, a decrease in the fluorescence signal of cells treated with SPIONs-CPTES and SPIONs-CPTES-HAPtS is observed compared to the untreated cells, which supposes that these nanoparticles could damage the plasma membrane. This result does not exclude the interference of nanoparticles with this kind of assay, as it is known for the colorimetric and fluorimetric tests. Indeed, Arenda et al. [61] reported that the aggregation of nanoparticles has an effect on the quenching of the fluorescein’s fluorescence. In order to illustrate this kind of interference, a fluorescence measurement was carried out for fluorescein with and without the presence of bare SPIONs (60 µg·mL^−1^) (Figure S7). The result shows the decrease of the fluorescence signal to half immediately after the SPIONs addition, which illustrates the capacity of nanoparticles in the quenching of FITC fluorescence. As the interaction of cells–nanoparticles depends on the latter’s surface chemistry, it is difficult to estimate the interference of the nanoparticles via this method. On the other hand, the MTT test evaluates the cells viability through assessing their metabolic activity (metabolically active mitochondria). Figure 10B shows that the quantity of cells remains almost the same for all studied samples. This suggests that the nanoparticles do not affect the mitochondrial activity. We should notice that the cell’s viability of 120%, observed for SPIONs-CPTES, could be explained by nanoparticle interference [62]. To conclude, the obtained results with FDA and MTT tests suggest a negative impact of the SPIONs-CPTES-HAPtS on the plasma membrane without any effect on the mitochondrial activity. This does not allow any clear conclusion regarding the effect of nanoparticles on cell viability, which led to performing the clonogenic test.

#### 3.3.2. Clonogenic Assay

**Figure 11:**
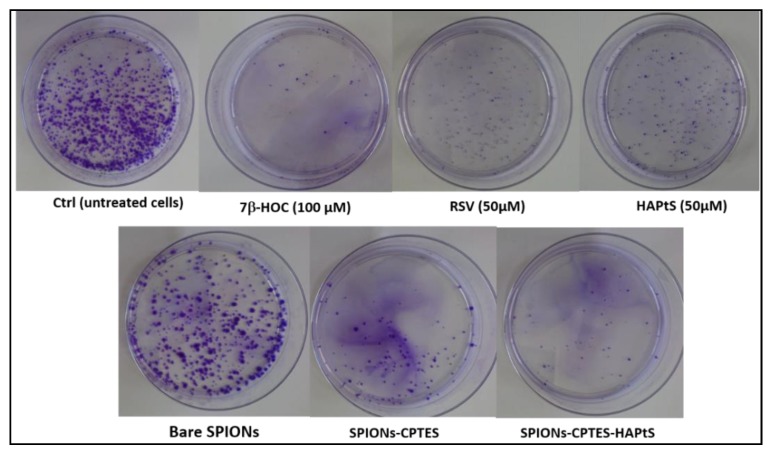
Pictures of Petri dishes of C6 cells at the end of the clonogenic assay (24 h of incubation with nanoparticles or molecules and 8 days of culture). Ctrl (untreated cells), positive cytotoxic control (7β-OHC, 100 µM), RSV (50 µM), HAPtS (50 µM in RSV equivalent), bare SPIONs (the same amount as in SPIONs-CPTES-HAPtS), SPIONs-CPTES (the same amount as in SPIONs-CPTES-HAPtS), and SPIONs-CPTES-HAPtS (amount equivalent to 50 µM HAPtS).

The clonogenic test evaluates the capacity of molecule/mixtures of molecules/nanoparticles to avoid the proliferation of cancerous cells after incubation for 24 h. This test permits assessment of the proliferation capacity of potentially viable cells (based on cells counting using trypan blue) after exposure to nanoparticles [28]. It was carried out on C6 rat glioma cells. The obtained results, shown in Figure 11, prove that RSV and HAPtS molecules have almost the same effect in reducing cell proliferation at a concentration of 50 µM, since they reduce the number and the size of colonies compared to control (untreated cells). On the other hand, bare SPIONs (500 µg·mL^−1^) do not show any significant anti-clonogenic effect, since a similar number of colonies is formed with almost the same size. However, cells treated with functionalized SPIONs lose their capacity of proliferation (colonies formation), which is illustrated by the small number of the developed colonies for SPIONs-CPTES and SPIONs-CPTES-HAPtS samples. The slight cytotoxic effect observed in this work, with FDA and clonogenic tests for SPIONs-CPTES, has been also observed in the study of Bollu et al. for CPTES-modified mesoporous silica nanoparticles [63]. This result could be explained by the presence of the chlorinated organic carbon chain in the CPTES molecule [64]. We can consider that the cytotoxic effect linked to the CPTES molecule in the SPIONs-CPTES-HAPtS sample is neglected, since the amount of chlorine is reduced to the half (according to XPS analysis) and HAPtS is the main molecule that is in direct contact with the cells. The number of C6 cell colonies is expressed as anti-clonogenic activity and the results are shown in Figure S8 (control value is set to 0%). The anti-clonogenic activity of SPIONs-CPTES-HAPtS is higher than that obtained for free RSV and HAPtS, which proves the HAPtS molecule has the best efficiency in limiting the proliferation of cancerous C6 cells when it is associated with SPIONs at the same concentration.

The results obtained with clonogenic assay could be correlated with FDA results showing a possible damage of the cellular membrane caused by SPIONs-CPTES and SPIONs-CPTES-HAPtS nanoparticles, as is shown in Figure 10, which could be the main cause of the cell’s incapacity to replicate. These preliminary results could be explained by the fact that SPIONs could: (i) favor the deregulation of localized signaling pathways at the level of plasma membrane involved in the life-death balance of the cells; (ii) contribute to better internalization of HAPtS inside the cell through endocytic vesicles [62,65]; (iii) increase the half-life of HAPtS in the cell by delaying its catabolism. These different points could contribute, at least in part, to the anti-proliferative and cytotoxic activity of HAPtS.

## 4. Conclusions

In this work, new nanohybrid materials based on superparamagnetic iron oxide nanoparticles have been elaborated to deliver a derivative of the RSV molecule, a natural compound known for its antioxidant activity, in order to elaborate new nanovectors for brain disease treatment. This molecule, HAPtS, was exclusively synthesized to be adapted for grafting onto SPIONs through an alkyl chain ending with a functional amine group. The evaluation of the antioxidant potential of this molecule by the ORAC test showed a similar antioxidant capacity to that obtained for RSV. The surface modification of SPIONs with HAPtS was carried out using the organosilane CPTES as a coupling agent and happened via a nucleophilic substitution reaction to yield 116 µmol of HAPtS per g of SPIONs, an amount estimated by thermogravimetric analysis. The elaborated nanohybrids were fully characterized by DLS and TEM images that show the evolution of the nanohybrids size and colloidal stability after the sequential grafting. The immobilized molecules grafted onto SPION’s surface were also analyzed using zeta potential measurement, IR spectra, and XPS analysis, which permits illustration of the evolution of the surface chemistry of SPIONs and confirms the presence of an organic shell. The biological efficiency of elaborated nanoparticles was assessed by MTT, FDA, and clonogenic tests. The results show that SPIONs-CPTES-HAPtS damage the plasma membrane of C6 rat glioma cells; this side effect could subsequently limit the cell proliferation of C6 cells, making SPIONs-HAPtS potential candidates for targeted cancer treatment, including the glioma. 

## Figures and Tables

**Table 1:** Experimental and calculated XPS elemental analysis of HAPtS.

Element (%)	C1s	O1s	N1s	Si2p	Ca2p	C/N
**Calculated**	85.0	10.0	5.0	-	-	17
**Experimental**	81.1	14.3	3.4	0.4	0.8	23

**Table 2:** Comparison between the calculated and the experimental proportion of the chemical bonds obtained from the fitting of C1s, O1s, and N1s XPS peaks.

Element	C1s			O1s		N1s
**Chemical bonds**	C-C/C-H	C-O/C-N	C=O	H-O-C	C-O-C	-NH_2_
**Calculated (%)**	76.5	23.5	-	50.0	50.0	100.0
**Experimental (%)**	70.0	24.0	6.0	54.0	46.0	100.0

**Table 3:** Physico-chemical characteristics of bare and modified SPIONs.

Parameter		Bare SPIONs	SPIONs-CPTES	SPIONs-CPTES- HAPtS
	**Isoelectric point**	8.3	5.9	8.2
**Hydrodynamic size in water**	**dH (number) (nm)**	21 ± 8	26 ± 7	92 ± 20
	**PDI**	0.157 ± 0.004	0.189 ± 0.003	0.329 ± 0.026
	**Z-average (nm)**	67 ± 1	96 ± 5	145 ± 10
**Hydrodynamic size in PBS** 0.1×	**dH (number) (nm)**	60 ± 9	50 ± 8	118 ± 25
	**PDI**	0.489 ± 0.016	0.230 ± 0.004	0.410 ± 0.012
	**Z-average (nm)**	299 ± 16	135 ± 12	220 ± 8
	**TEM measured diameter (nm)**	9.0 ± 2.0	8.7 ± 1.5	9.6 ± 1.6

**Table 4:** XPS elemental analysis of bare and functionalized SPIONs (atomic percentage).

Element (%)	C1s	O1s	Fe2p	N1s	Si2p	Cl2p	Cl/Si
**Bare SPIONs**	3.0	57.5	38.4	1.1	-	-	-
Element SPIONs/Fe	0.08	1.50	1.00	0.03			
**SPIONs-CPTES**	15.2	49.1	28.2	-	5.3	2.2	0.4
Element SPIONs-CPTES/Fe	0.54	1.74	1.00	-	0.19	0.07	-
**SPIONs-CPTES-HAPtS**	21.6	48.0	24.7	0.4	4.2	1.1	0.2
Element SPIONs-CPTES-HAPtS/Fe	0.87	1.94	1.00	0.02	0.17	0.04	-

**Table 5:** Thermogravimetric data and calculation of the number of molecules on SPIONs’ surface.

Samples	Weight Loss (%)	Degraded Molecule during Heating		Compounds on SPIONs Surface	
		Chemical Formula	Molecular Weight (g·mol^−1^)	molecule·nm^−^²	µmol·g^−1^ SPIONs
Bare SPIONs	3.5	H_2_O	18	10.3 (OH)	1944 (OH)
SPIONs-CPTES	2.6	-(CH_2_)_3_-Cl	78	1.8 (CPTES)	340 (CPTES)
SPIONs-CPTES-HAPtS	3.6	-(CH_2_)_3_-NH-C_17_H_17_O_2_	310	0.6 (HAPtS)	116 (HAPtS)

## Supplementary Materials

The following are available online at https://www.mdpi.com/2079-4991/9/2/287/s1, Figure S1: XRD pattern of bare SPIONs (λ = 1.540598 Å). Figure S2: Photo showing the difference in the colloidal stability between bare SPIONs, SPIONs-CPTES, and SPIONs-HAPtS samples in PBS medium after 24 h (pH 7.4, 100 µg/mL of nanoparticles concentration). Figure S3: Hydrodynamic size vs. time of bare SPIONs, SPIONs-CPTES, and SPIONs-CPTES-HAPtS in PBS medium with bovine serum albumin (BSA, 40 g/L) at 37 °C. Figure S4: IR spectra of bare and functionalized SPIONs. Figure S5: XPS spectrum of N1s peak for SPIONs-HAPtS sample. Figure S6. Derivative of TGA curves of bare SPIONs, SPIONs-CPTES, and SPIONs-CPTES-HAPtS. Figure S7: Excitation and emission spectra of fluorescein with and without the presence of bare SPIONs showing the fluorescence quenching effect of SPIONs at the concentration 60 µg·mL^−1^ in PBS 0.1×. Figure S8: Anti-clonogenic activity (expressed in %, control value is set to 0%) on C6 cells after 24 h of incubation with nanoparticles or molecules and 8 days of culture. Ctrl, untreated cells, positive cytotoxic control (7β-OHC, 100 µM), RSV (50 µM), HAPtS (50 µM), bare SPIONs (the same amount as in SPIONs-CPTES-HAPtS), SPIONs-CPTES (the same amount as in SPIONs-CPTES-HAPtS), and SPIONs-CPTES-HAPtS (amount equivalent to 50 µM HAPtS).

## References

[1] Wenzel E., Somoza V. (2005). Metabolism and bioavailability of trans-resveratrol. Mol. Nutr. Food Res..

[2] Siemann E., Creasy L. (1992). Concentration of the phytoalexin resveratrol in wine. Am. J. Enol. Vitic..

[3] Frankel E., German J., Kinsella J., Parks E., Kanner J. (1993). Inhibition of oxidation of human low-density lipoprotein by phenolic substances in red wine. Lancet.

[4] Stivala L.A., Savio M., Carafoli F., Perucca P., Bianchi L., Maga G., Forti L., Pagnoni U.M., Albini A., Prosperi E. (2001). Specific structural determinants are responsible for the antioxidant activity and the cell cycle effects of resveratrol. J. Boil. Chem..

[5] Chalal M., Klinguer A., Echairi A., Meunier P., Vervandier-Fasseur D., Adrian M. (2014). Antimicrobial activity of resveratrol analogues. Molecules.

[6] Tili E., Michaille J.-J., Adair B., Alder H., Limagne E., Taccioli C., Ferracin M., Delmas D., Latruffe N., Croce C.M. (2010). Resveratrol decreases the levels of miR−155 by upregulating miR-663, a microRNA targeting JunB and JunD. Carcinogenesis.

[7] Aggarwal B.B., Bhardwaj A., Aggarwal R.S., Seeram N.P., Shishodia S., Takada Y. (2004). Role of resveratrol in prevention and therapy of cancer: Preclinical and clinical studies. Anticancer Res..

[8] Holmes-McNary M., Baldwin A.S. (2000). Chemopreventive properties of trans-resveratrol are associated with inhibition of activation of the IκB kinase. Cancer Res..

[9] Athar M., Back J.H., Tang X., Kim K.H., Kopelovich L., Bickers D.R., Kim A.L. (2007). Resveratrol: A review of preclinical studies for human cancer prevention. Toxicol. Appl. Pharmacol..

[10] Perez-Campo R., López-Torres M., Cadenas S., Rojas C., Barja G. (1998). The rate of free radical production as a determinant of the rate of aging: Evidence from the comparative approach. J. Comp. Physiol. B.

[11] Beckman K.B., Ames B.N. (1998). The Free Radical Theory of Aging Matures. Physiol. Rev..

[12] Sun A.Y., Wang Q., Simonyi A., Sun G.Y. (2010). Resveratrol as a Therapeutic Agent for Neurodegenerative Diseases. Mol. Neurobiol..

[13] Ranney A., Petro M.S. (2009). Resveratrol protects spatial learning in middle-aged C57BL/6 mice from effects of ethanol. Behav. Pharmacol..

[14] Wang Q., Xu J., Rottinghaus G.E., Simonyi A., Lubahn D., Sun G.Y., Sun A.Y. (2002). Resveratrol protects against global cerebral ischemic injury in gerbils. Brain Res..

[15] Kim D., Nguyen M.D., Dobbin M.M., Fischer A., Sananbenesi F., Rodgers J.T., Delalle I., Baur J.A., Sui G., Armour S.M. (2007). SIRT1 deacetylase protects against neurodegeneration in models for Alzheimer’s disease and amyotrophic lateral sclerosis. EMBO J..

[16] Kuhnle G., Spencer J.P., Chowrimootoo G., Schroeter H., Debnam E.S., Srai S.K.S., Rice-Evans C., Hahn U. (2000). Resveratrol is absorbed in the small intestine as resveratrol glucuronide. Biochem. Biophys. Res. Commun..

[17] Bertelli A., Giovannini L., Stradi R., Urien S., Tillement J., Bertelli A. (1996). Kinetics of trans-and cis-resveratrol (3,4′,5-trihydroxystilbene) after red wine oral administration in rats. Int. J. Clin. Pharmacol. Res..

[18] Summerlin N., Soo E., Thakur S., Qu Z., Jambhrunkar S., Popat A. (2015). Resveratrol nanoformulations: Challenges and opportunities. Int. J. Pharm..

[19] Spigno G., Donsì F., Amendola D., Sessa M., Ferrari G., De Faveri D.M. (2013). Nanoencapsulation systems to improve solubility and antioxidant efficiency of a grape marc extract into hazelnut paste. J. Food Eng..

[20] Lee C.-W., Yen F.-L., Huang H.-W., Wu T.-H., Ko H.-H., Tzeng W.-S., Lin C.-C. (2012). Resveratrol nanoparticle system improves dissolution properties and enhances the hepatoprotective effect of resveratrol through antioxidant and anti-inflammatory pathways. J. Agric. Food Chem..

[21] Isailović B.D., Kostić I.T., Zvonar A., Đorđević V.B., Gašperlin M., Nedović V.A., Bugarski B.M. (2013). Resveratrol loaded liposomes produced by different techniques. Innov. Food Sci. Emerg. Technol..

[22] Blond J., Denis M., Bezard J. (1995). Antioxidant action of resveratrol in lipid peroxidation. Sci. Aliment. (France).

[23] Pando D., Gutiérrez G., Coca J., Pazos C. (2013). Preparation and characterization of niosomes containing resveratrol. J. Food Eng..

[24] Pando D., Caddeo C., Manconi M., Fadda A.M., Pazos C. (2013). Nanodesign of olein vesicles for the topical delivery of the antioxidant resveratrol. J. Pharm. Pharmacol..

[25] Peng H., Xiong H., Li J., Xie M., Liu Y., Bai C., Chen L. (2010). Vanillin cross-linked chitosan microspheres for controlled release of resveratrol. Food Chem..

[26] Kim S., Ng W.K., Dong Y., Das S., Tan R.B. (2012). Preparation and physicochemical characterization of trans-resveratrol nanoparticles by temperature-controlled antisolvent precipitation. J. Food Eng..

[27] Wang M., Li L., Zhang X., Liu Y., Zhu R., Liu L., Fang Y., Gao Z., Gao D. (2018). Magnetic Resveratrol Liposomes as a New Theranostic Platform for Magnetic Resonance Imaging Guided Parkinson’s Disease Targeting Therapy. ACS Sustain. Chem. Eng..

[28] Shukla S.P., Roy M., Mukherjee P., Das L., Neogy S., Srivastava D., Adhikari S. (2016). Size Selective Green Synthesis of Silver and Gold Nanoparticles: Enhanced Antibacterial Efficacy of Resveratrol Capped Silver Sol. J. Nanosci. Nanotechnol..

[29] Park S., Cha S.-H., Cho I., Park S., Park Y., Cho S., Park Y. (2016). Antibacterial nanocarriers of resveratrol with gold and silver nanoparticles. Mater. Sci. Eng. C.

[30] Ganesh Kumar C., Poornachandra Y., Mamidyala S.K. (2014). Green synthesis of bacterial gold nanoparticles conjugated to resveratrol as delivery vehicles. Colloids Surf. B Biointerfaces.

[31] Laurent S., Forge D., Port M., Roch A., Robic C., Vander Elst L., Muller R.N. (2008). Magnetic Iron Oxide Nanoparticles: Synthesis, Stabilization, Vectorization, Physicochemical Characterizations, and Biological Applications. Chem. Rev..

[32] Canet E., Revel D., Forrat R., Baldy-Porcher C., de Lorgeril M., Sebbag L., Vallee J.-P., Didier D., Amiel M. (1993). Superparamagnetic iron oxide particles and positive enhancement for myocardial perfusion studies assessed by subsecond T1-weighted MRI. Magn. Reson. Imaging.

[33] Thomas G., Demoisson F., Chassagnon R., Popova E., Millot N. (2016). One-step continuous synthesis of functionalized magnetite nanoflowers. Nanotechnology.

[34] Gupta A.K., Gupta M. (2005). Synthesis and surface engineering of iron oxide nanoparticles for biomedical applications. Biomaterials.

[35] Perez J.M., Josephson L., Weissleder R. (2004). Use of Magnetic Nanoparticles as Nanosensors to Probe for Molecular Interactions. ChemBioChem.

[36] Arruebo M., Fernández-Pacheco R., Ibarra M.R., Santamaría J. (2007). Magnetic nanoparticles for drug delivery. Nano Today.

[37] Saraiva C., Praça C., Ferreira R., Santos T., Ferreira L., Bernardino L. (2016). Nanoparticle-mediated brain drug delivery: Overcoming blood–brain barrier to treat neurodegenerative diseases. J. Control. Release.

[38] Champagne P.-O., Westwick H., Bouthillier A., Sawan M. (2018). Colloidal stability of superparamagnetic iron oxide nanoparticles in the central nervous system: A review. Nanomedicine.

[39] Chertok B., Moffat B.A., David A.E., Yu F., Bergemann C., Ross B.D., Yang V.C. (2008). Iron oxide nanoparticles as a drug delivery vehicle for MRI monitored magnetic targeting of brain tumors. Biomaterials.

[40] Chertok B., David A.E., Yang V.C. (2010). Polyethyleneimine-modified iron oxide nanoparticles for brain tumor drug delivery using magnetic targeting and intra-carotid administration. Biomaterials.

[41] Tabatabaei S.N., Girouard H., Carret A.-S., Martel S. (2015). Remote control of the permeability of the blood–brain barrier by magnetic heating of nanoparticles: A proof of concept for brain drug delivery. J. Control. Release.

[42] Dan M., Cochran D.B., Yokel R.A., Dziubla T.D. (2013). Binding, Transcytosis and Biodistribution of Anti-PECAM−1 Iron Oxide Nanoparticles for Brain-Targeted Delivery. PLoS ONE.

[43] Chalal M., Delmas D., Meunier P., Latruffe N., Vervandier-Fasseur D. (2014). Inhibition of cancer derived cell lines proliferation by synthesized hydroxylated stilbenes and new ferrocenyl-stilbene analogs. Comparison with resveratrol. Molecules.

[44] Daubresse N., Francesch C., Rolando C. (1998). Phase transfer Wittig reaction with 1,3-dioxolan-2-yl-methyltriphenyl phosphonium salts: An efficient method for vinylogation of aromatic aldehydes. Tetrahedron.

[45] Sabale S., Kandesar P., Jadhav V., Komorek R., Motkuri R.K., Yu X.-Y. (2017). Recent developments in the synthesis, properties, and biomedical applications of core/shell superparamagnetic iron oxide nanoparticles with gold. Biomater. Sci..

[46] Nury T., Zarrouk A., Ragot K., Debbabi M., Riedinger J.-M., Vejux A., Aubourg P., Lizard G. (2017). 7-Ketocholesterol is increased in the plasma of X-ALD patients and induces peroxisomal modifications in microglial cells: Potential roles of 7-ketocholesterol in the pathophysiology of X-ALD. J. Steroid Biochem. Mol. Boil..

[47] Franken N.A.P., Rodermond H.M., Stap J., Haveman J., van Bree C. (2006). Clonogenic assay of cells in vitro. Nat. Protoc..

[48] Lizard G., Gueldry S., Deckert V., Gambert P., Lagrost L. (1997). Evaluation of the cytotoxic effects of some oxysterols and of cholesterol on endothelial cell growth: Methodological aspects. Pathol. Biol. (Paris).

[49] Garvey M., Moriceau B., Passow U. (2007). Applicability of the FDA assay to determine the viability of marine phytoplankton under different environmental conditions. Mar. Ecol. Prog. Ser..

[50] Socrates G. (2004). Infrared and Raman Characteristic Group Frequencies: Tables and Charts.

[51] Zhou Z., Li W., Sun W.-J., Lu T., Tong H.H., Sun C.C., Zheng Y. (2016). Resveratrol cocrystals with enhanced solubility and tabletability. Int. J. Pharm..

[52] Trela B.C., Waterhouse A.L. (1996). Resveratrol:  Isomeric Molar Absorptivities and Stability. J. Agric. Food Chem..

[53] López-Nicolás J.M., García-Carmona F. (2008). Aggregation state and p K a values of (E)-resveratrol as determined by fluorescence spectroscopy and UV− visible absorption. J. Agric. Food Chem..

[54] Thomas G., Demoisson F., Boudon J., Millot N. (2016). Efficient functionalization of magnetite nanoparticles with phosphonate using a one-step continuous hydrothermal process. Dalton Trans..

[55] Guigue-Millot N., Champion Y., Hÿtch M.J., Bernard F., Bégin-Colin S., Perriat P. (2001). Chemical Heterogeneities in Nanometric Titanomagnetites Prepared by Soft Chemistry and Studied Ex Situ:  Evidence for Fe-Segregation and Oxidation Kinetics. J. Phys. Chem. B.

[56] Perriat P., Fries E., Millot N., Domenichini B. (1999). XPS and EELS investigations of chemical homogeneity in nanometer scaled Ti-ferrites obtained by soft chemistry. Solid State Ion..

[57] Sallem F., Boudon J., Heintz O., Séverin I., Megriche A., Millot N. (2017). Synthesis and characterization of chitosan-coated titanate nanotubes: Towards a new safe nanocarrier. Dalton Trans..

[58] Jesionowski T., Ciesielczyk F., Krysztafkiewicz A. (2010). Influence of selected alkoxysilanes on dispersive properties and surface chemistry of spherical silica precipitated in emulsion media. Mater. Chem. Phys..

[59] Ishida H., Koenig J.L. (1978). Fourier transform infrared spectroscopic study of the structure of silane coupling agent on E-glass fiber. J. Colloid Interface Sci..

[60] Chuang W., Geng-sheng J., Lei P., Bao-lin Z., Ke-zhi L., Jun-long W. (2018). Influences of surface modification of nano-silica by silane coupling agents on the thermal and frictional properties of cyanate ester resin. Results Phys..

[61] Aranda A., Sequedo L., Tolosa L., Quintas G., Burello E., Castell J.V., Gombau L. (2013). Dichloro-dihydro-fluorescein diacetate (DCFH-DA) assay: A quantitative method for oxidative stress assessment of nanoparticle-treated cells. Toxicol. Vitro.

[62] Maurizi L., Papa A.-L., Dumont L., Bouyer F., Walker P., Vandroux D., Millot N. (2015). Influence of surface charge and polymer coating on internalization and biodistribution of polyethylene glycol-modified iron oxide nanoparticles. J. Biomed. Nanotechnol..

[63] Bollu V.S., Barui A.K., Mondal S.K., Prashar S., Fajardo M., Briones D., Rodríguez-Diéguez A., Patra C.R., Gómez-Ruiz S. (2016). Curcumin-loaded silica-based mesoporous materials: Synthesis, characterization and cytotoxic properties against cancer cells. Mater. Sci. Eng. C.

[64] Geerlings P., Tafazoli M., Kirsch-Volders M., Baeten A. (1998). In vitro mutagenicity and genotoxicity study of a number of short-chain chlorinated hydrocarbons using the micronudeus test and the alkaline single cell gel electrophoresis technique (Comet assay) in human lymphocytes: A structure–activity relationship (QSAR) analysis of the genotoxic and cytotoxic potential. Mutagenesis.

[65] Sruthi S., Maurizi L., Nury T., Sallem F., Boudon J., Riedinger J.M., Millot N., Bouyer F., Lizard G. (2018). Cellular interactions of functionalized superparamagnetic iron oxide nanoparticles on oligodendrocytes without detrimental side effects: Cell death induction, oxidative stress and inflammation. Colloids Surf. B Biointerfaces.

