# Supplementary material for: Elaboration of Trans-Resveratrol Derivative-Loaded Superparamagnetic Iron Oxide Nanoparticles for Glioma Treatment
Source: Nanomaterials (Basel). 2019 Feb 18;9(2):287. doi: 10.3390/nano9020287 (PMC6409721; doi:10.3390/nano9020287)
Supplement: Supplementary file 1 [file nanomaterials-09-00287-s001.pdf]

nanomaterials

Article

Elaboration of *Trans*-Resveratrol Derivative-Loaded Superparamagnetic Iron Oxide Nanoparticles for Glioma Treatment

Fadoua Sallem [1], Rihab Haji [2], Dominique Vervandier-Fasseur [2], Thomas Nury [3], Lionel Maurizi [1], Julien Boudon [1], Gérard Lizard [3] and Nadine Millot [1,*]

[1] Laboratoire Interdisciplinaire Carnot de Bourgogne (ICB), UMR 6303 CNRS/Université Bourgogne Franche-Comté, 21000 Dijon, France ; fadouasallem@gmail.com (F.S.); lionelmaurizi@gmail.com (L.M.); julien.boudon@u-bourgogne.fr (J.B.)

[2] Institut de Chimie Moléculaire de l'Université de Bourgogne (ICMUB), UMR 6302 CNRS/Université Bourgogne Franche-Comté, 21000 Dijon, France ; hajirihab22@yahoo.com (R.H.); dominique.vervandier-fasseur@u-bourgogne.fr (D.V.-F.)

[3] Laboratoire Bio-PeroxIL, EA7270, Université de Bourgogne Franche-Comté/Inserm, 21000 Dijon, France ; thomas.nury@u-bourgogne.fr (T.N.);gerard.lizard@u-bourgogne.fr (G.L.)

* Correspondence: nadine.millot@u-bourgogne.fr

Supplementary materials

Contents:

Supplementary materials SM1: Additional experimental details and NMR data of the organic synthesis of 4′-hydroxy-4-(3-aminopropoxy) *trans*-stilbene (HAPtS)

Figure S1. XRD pattern of bare SPIONs (λ = 1.540598 Å).

Figure S2. Photo showing the difference in the colloidal stability between bare SPIONs, SPIONs-CPTES and SPIONs-CPTES-HAPtS samples in PBS medium after 24 h (pH 7.4, 100 µg/mL of nanoparticles concentration).

Figure S3. Hydrodynamic size vs. time of bare SPIONs, SPIONs-CPTES and SPIONs-CPTES-HAPtS in PBS medium with bovine serum albumin (BSA, 40 g/L) at 37 °C.

Figure S4. IR spectra of bare and functionalized SPIONs.

Figure S5. XPS spectrum of N1s peak for SPIONs-CPTES-HAPtS sample.

Supplementary materials SM2: Details of grafting rate calculation using TGA data

Figure S6. Derivative of TGA curves of bare SPIONs, SPIONs-CPTES and SPIONs-CPTES-HAPtS.

Figure S7. Excitation and emission spectra of fluorescein with and without the presence of bare SPIONs showing the fluorescence quenching effect of SPIONs at the concentration 60 µg·mL^{-1} in PBS 10^{-3} M.

Figure S81. Anti-clonogenic activity (expressed in %, control value is set to 0%) on C6 cells after 24 h of incubation with nanoparticles / molecules and 8 days of culture. Ctrl; untreated cells, positive cytotoxic control (7β-OHC, 100 μM), RSV (50 μM), HAPtS (50 μM), bare SPIONs (the same amount as in SPIONs-CPTES-HAPtS), SPIONs-CPTES (the same amount as in SPIONs-CPTES-HAPtS) and SPIONs-CPTES-HAPtS (amount equivalent to 50 μM HAPtS).

Supplementary materials SM1: Additional experimental details and NMR data of the organic synthesis of 4′-hydroxy-4-(3-aminopropoxy) *trans*-stilbene (HAPtS)

4′-hydroxy-4-(3-aminopropoxy)trans-stilbene was synthesized by a Wittig reaction from 4-acetoxybenzyltriphenylphosphonium and N-3-(4-carbaldehydephenoxy)propylphtalimide. The Wittig reaction was carried out in phase transfer conditions [1].

Synthesis of 4-acetoxybenzyltriphenylphosphonium (A)

The phosphonium bromide was prepared in three steps from 4-hydroxybenzylic alcohol [2].

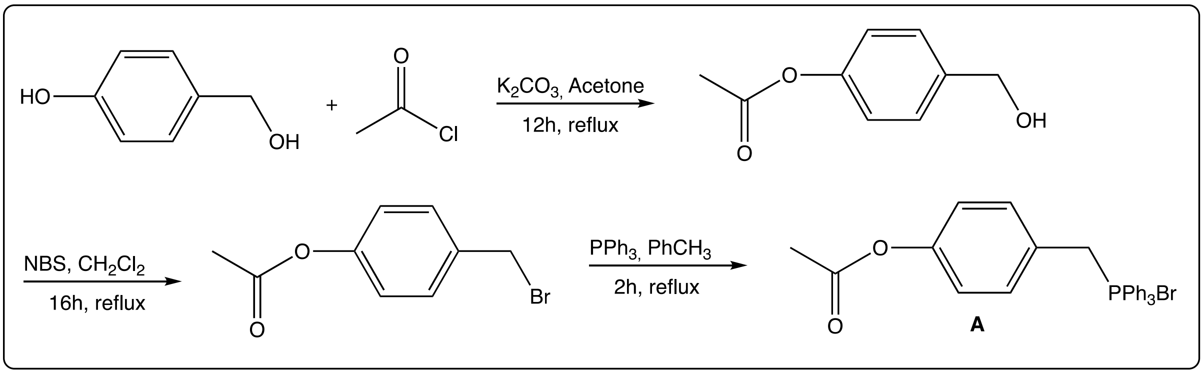

NMR ^{1}H (CDCl$_3$, 300 MHz)

δ (ppm): 2.26 (s, 3H); 5.45 (d, 2H); 6.8 (d, 2H); 7,2 (d, 2H); 7.62-7.79 (m, 15H)

Synthesis of N-3-(4-carbaldehydephenoxy)propylphtalimide (B)

4-hydroxybenzaldehyde was reacted with N-(3-bromopropyl)phtalimide (1 equivalent) in the presence of K_2CO_3 (1,5 equivalent) in refluxed acetone during 16 hours. After filtration of the inorganic salts, the solvent was removed. The residue was dissolved in methylene chloride and the organic solution was washed with water and brine. After removal the solvent, the residue was crystallized from ethanol, yield 56%.

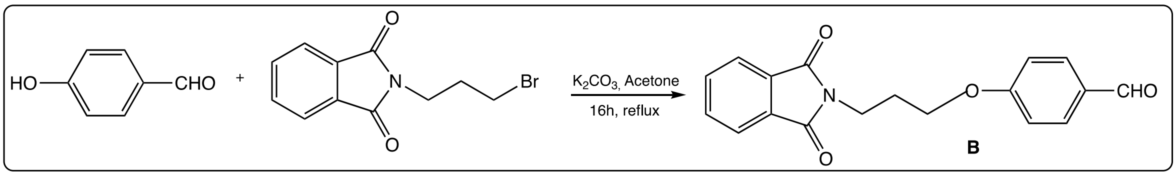

NMR ^{1}H (CDCl$_3$, 300 MHz)

δ (ppm): 2.55 (quint, 2H); 3.93 (t, 2H); 4.10 (d, 2H); 6.76-6.94 (m, 4H); 7.66-7.95 (m, 4H); 9.87 (s, 1H)

Synthesis of 4-acetoxy-4′-N-(3-O-propylphtalimide)trans-stilbene (C)

A saturated aqueous solution of K_2CO_3 (10 mL), TDA-1 (tris-(3,6-dioxaheptyl)amine, 0.5 mL) and methylene chloride (10 mL) were vigorously stirred. Phosphonium bromide (1 equivalent) and aldehyde (1 equivalent) were successively added to the previous suspension. The mixture was magnetically stirred at room temperature, overnight. After the work-up, the stilbene was recrystallized from ethanol, yield 8%.

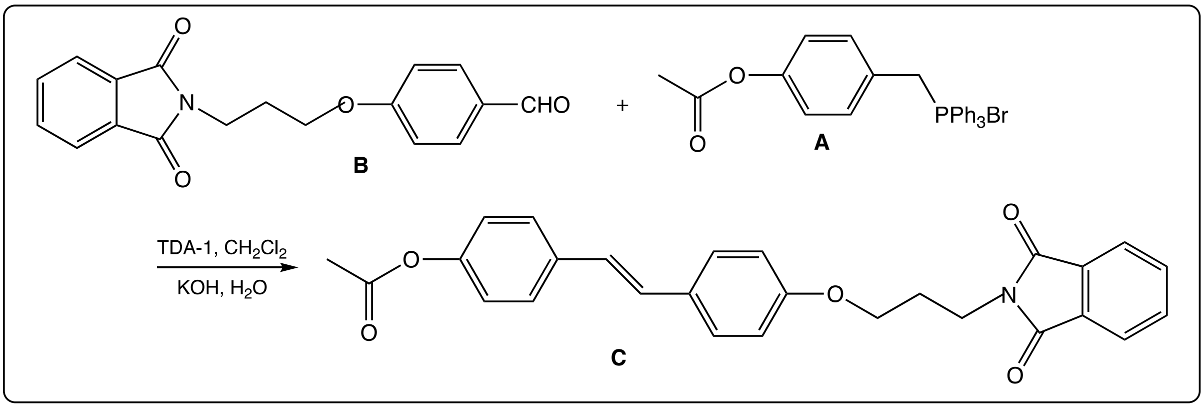

NMR ^{1}H ($CDCl_3$, 300 MHz)
δ (ppm): 2.14-2.26 (q, 2H); 2.30 (s, 3H); 3.88 (t, 2H); 4.06 (t, 2H); 6.73 (d, 2H); 6.83 (d, 2H); 6.86 (d, 2H); 7.11 (d, 2H); 7.33(d, 2H); 7.53 (d, 2H); 7.66 (d, 2H); 7.92 (d, 2H)

Synthesis of 4′-hydroxy-4-(3-aminopropoxy)trans-stilbene

To a suspension of protected stilbene (66 mg) in MeOH (6 mL), solid KOH (84 mg) was added. The mixture was stirred 2 hours at 40 °C, then hydrolyzed with water (30 mL) and still stirred for 4 hours. After removal of MeOH, the solution was acidified to pH = 7 by adding HCl 0.1 M solution. The de-protected stilbene was precipitated and filtrated, yield 82%

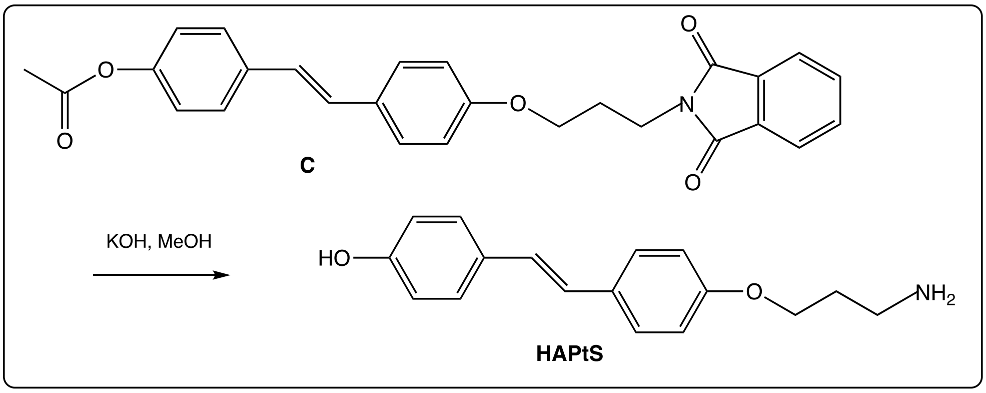

NMR ^{1}H ($CDCl_3$, 300 MHz)
δ (ppm): 1.3-1.44 (q, 2H); 2.76 (t, 3H); 3.40 (t, 2H); 6.06 (d, 2H); 6.25-6.79 (m, 8H); 6.98 (t, 2H); 8.58 (s, 1H)

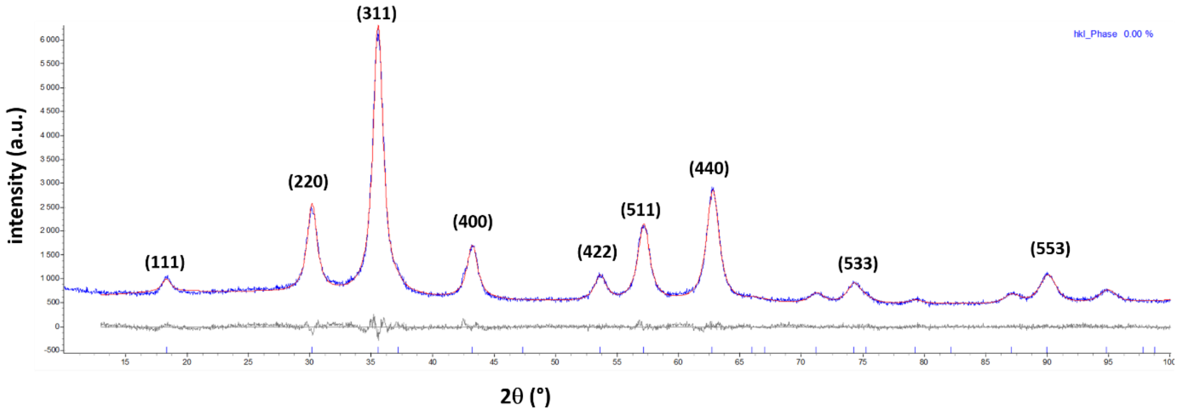

Figure S1. XRD pattern of bare SPIONs (λ = 1.540598 Å).

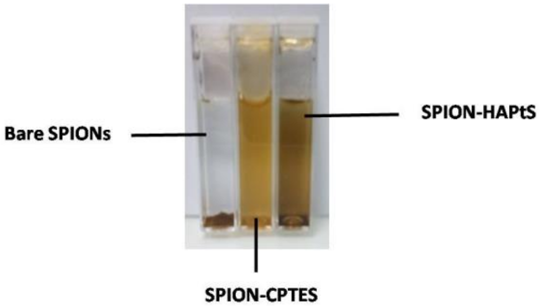

Figure S2. Photo showing the difference in the colloidal stability between bare SPIONs, SPIONs-CPTES and SPIONs-CPTES-HAPtS samples in PBS medium after 24 h (pH 7.4, 100 µg/mL of nanoparticles concentration).

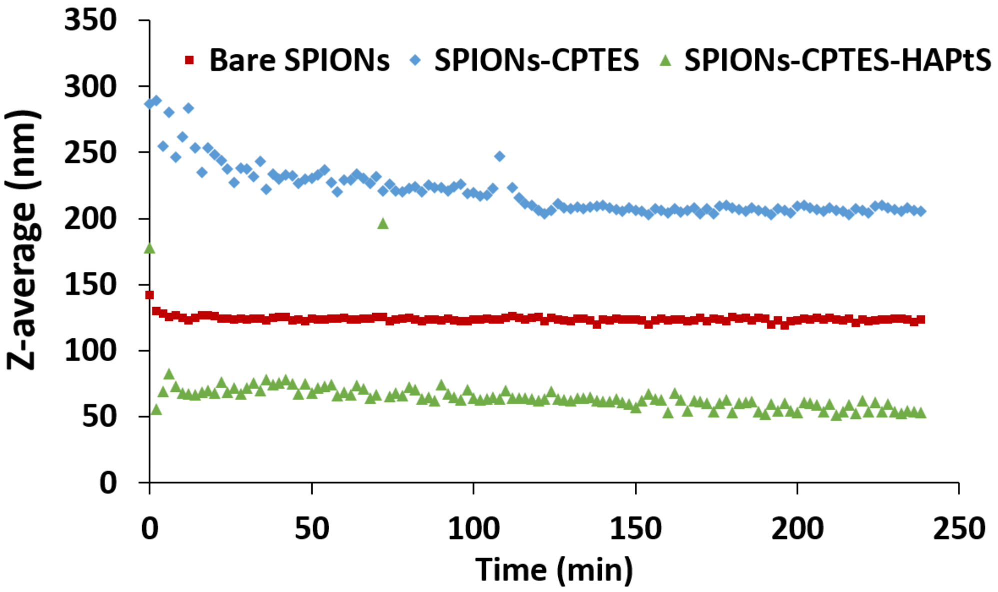

Figure S3. Hydrodynamic size vs. time of bare SPIONs, SPIONs-CPTES and SPIONs-CPTES-HAPtS in PBS medium with bovine serum albumin (BSA, 40 g/L) at 37 °C.

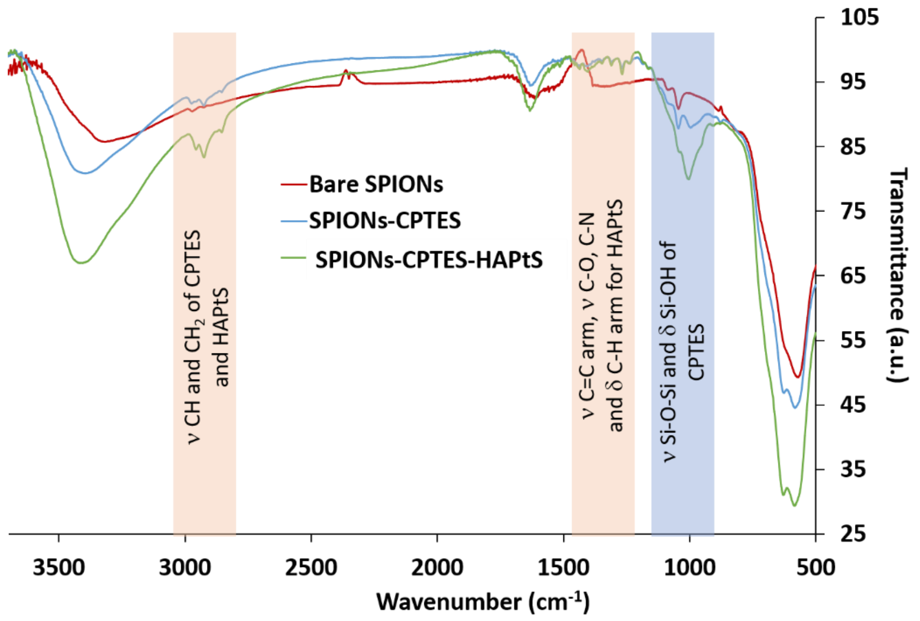

Figure S4. IR spectra of bare and functionalized SPIONs.

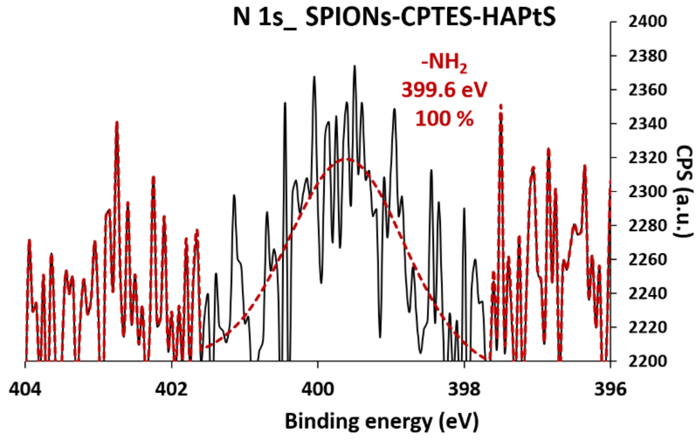

Figure S5. XPS spectrum of N1s peak for SPIONs-CPTES-HAPtS sample.

Supplementary materials SM2: Details of grafting rate calculation using TGA data:

In order to estimate the amount of grafted molecule (molecule.nm^{-2}), the following formulas were used:

$$\text{Grafting rate}\ \left(\text{molecule}\cdot\text{nm}^{-2}\right) = \frac{\Delta m/m \times N_A}{M \times S \times 10^{18}}\ (1)$$

$$\text{Hydroxyl amount}\ \left(\text{OH}\cdot\text{nm}^{-2}\right) = \frac{2 \times \Delta m/m \times N_A}{18 \times S \times 10^{18}}\ (2)$$

For the determination of the grafting rate in µmol.g^{-1} SPIONs, the following formulas were used:

$$\text{Grafting rate (µmol molecule}\cdot\text{g}^{-1}\ \text{SPIONs)} = \frac{\Delta m/m \times 10^6}{M}\ (3)$$

$$\text{Hydroxyl amount}\ \left(\text{µmol OH}\cdot\text{g}^{-1}\ \text{SPIONs}\right) = \frac{2 \times \Delta m/m \times 10^6}{18}\ (4)$$

Where $\Delta m/m$: is the weight loss (%), N_A: Avogadro constant 6.022 ×10^{23} (mol^{-1}), M: molecular weight of the decomposed molecule during the heat treatment (g/mol) and (S ×10^{18}): the specific surface area of bare or grafted SPIONs ($nm^2 \cdot g^{-1}$).

In the case of bare SPIONs, formula (2) is used to calculate the amount of hydroxyl groups onto SPIONs surface. Where M = 18 $g \cdot mol^{-1}$, the molecular weight of water, because it is the only lost molecule during the heat treatment of bare SPIONs. So, to lose one molecule of water, two hydroxyl groups are needed for the dehydroxylation process, therefore the factor two is added to the formula (2) compared to the formula (1).

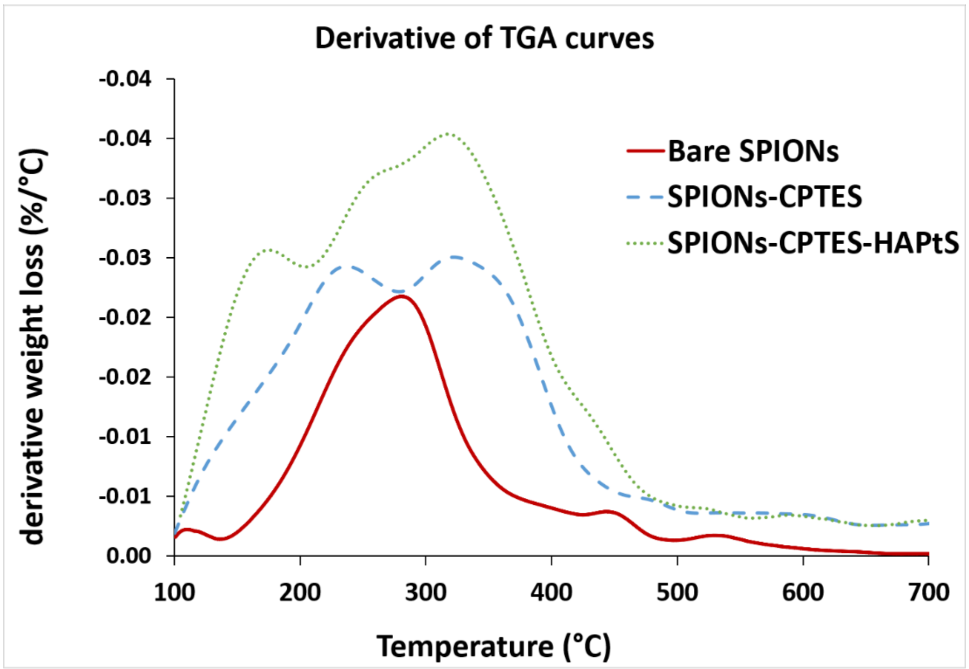

Figure S6. Derivative of TGA curves of bare SPIONs, SPIONs-CPTES and SPIONs-CPTES-HAPtS.

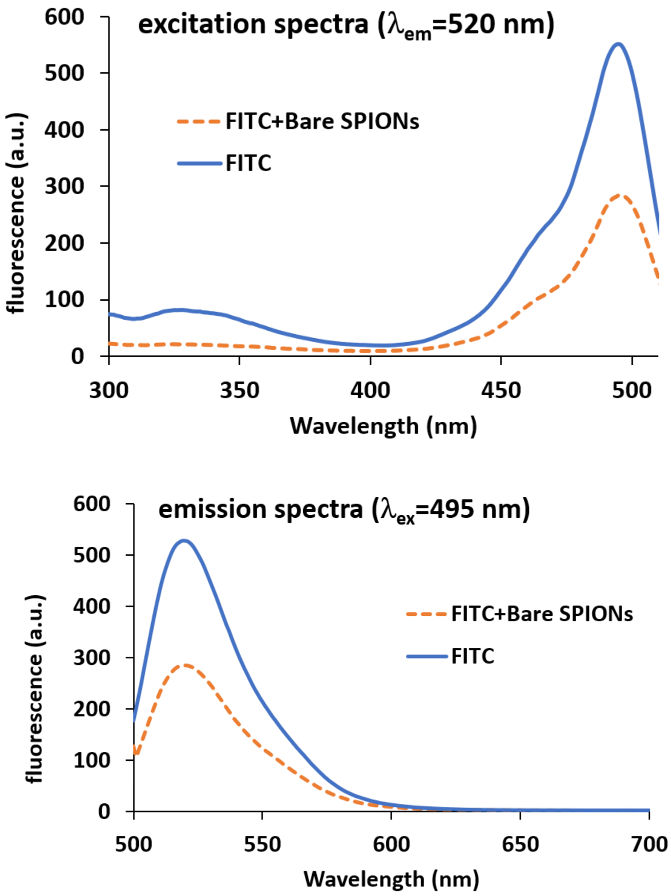

Figure S7. Excitation and emission spectra of fluorescein with and without the presence of bare SPIONs showing the fluorescence quenching effect of SPIONs at the concentration 60 µg·mL^{-1} in PBS 10^{-3} M.

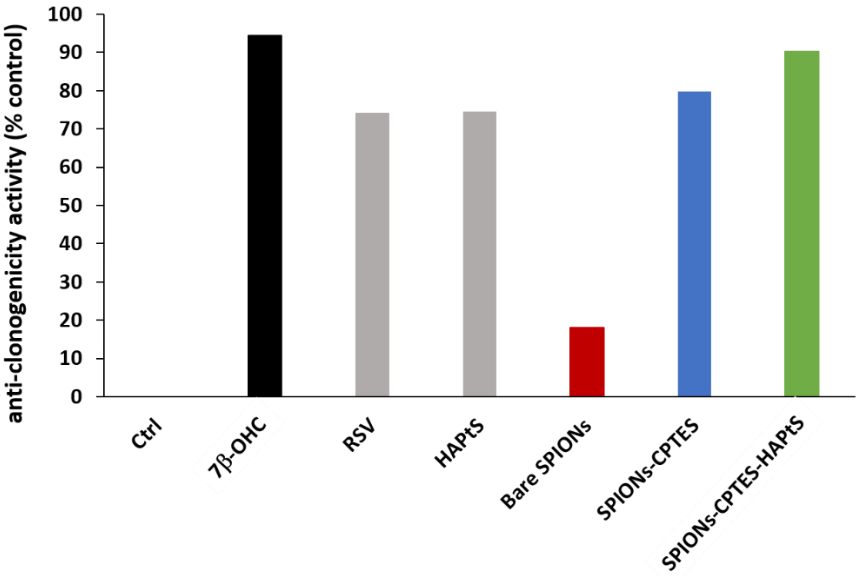

Figure S8. Anti-clonogenic activity (expressed in %, control value is set to 0%) on C6 cells after 24 h of incubation with nanoparticles/molecules and 8 days of culture. Ctrl; untreated cells, positive cytotoxic control (7β-OHC, 100 µM), RSV (50 µM), HAPtS (50 µM), bare SPIONs (the same amount

as in SPIONs-CPTES-HAPtS), SPIONs-CPTES (the same amount as in SPIONs-CPTES-HAPtS) and SPIONs-CPTES-HAPtS (amount equivalent to 50 μM HAPtS).

1. Daubresse, N.; Francesch, C.; Rolando, C. Phase transfer Wittig reaction with 1,3-dioxolan-2-yl-methyltriphenyl phosphonium salts: An efficient method for vinylogation of aromatic aldehydes. *Tetrahedron* **1998**, *54*, 10761–10770, doi:https://doi.org/10.1016/S0040-4020(98)00631-0.
2. Chalal, M.; Delmas, D.; Meunier, P.; Latruffe, N.; Vervandier-Fasseur, D. Inhibition of cancer derived cell lines proliferation by synthesized hydroxylated stilbenes and new ferrocenyl-stilbene analogs. Comparison with resveratrol. *Molecules* **2014**, *19*, 7850–7868.
